# Regional endothermy as a trigger for gigantism in some extinct macropredatory sharks

**DOI:** 10.1371/journal.pone.0185185

**Published:** 2017-09-22

**Authors:** Humberto G. Ferrón

**Affiliations:** Institut Cavanilles de Biodiversitat I Biologia Evolutiva, University of Valencia, Burjassot, Spain; University of California, UNITED STATES

## Abstract

Otodontids include some of the largest macropredatory sharks that ever lived, the most extreme case being *Otodus (Megaselachus) megalodon*. The reasons underlying their gigantism, distribution patterns and extinction have been classically linked with climatic factors and the evolution, radiation and migrations of cetaceans during the Paleogene. However, most of these previous proposals are based on the idea of otodontids as ectothermic sharks regardless of the ecological, energetic and body size constraints that this implies. Interestingly, a few recent studies have suggested the possible existence of endothermy in these sharks thus opening the door to a series of new interpretations. Accordingly, this work proposes that regional endothermy was present in otodontids and some closely related taxa (cretoxyrhinids), playing an important role in the evolution of gigantism and in allowing an active mode of live. The existence of regional endothermy in these groups is supported here by three different approaches including isotopic-based approximations, swimming speed inferences and the application of a novel methodology for assessing energetic budget and cost of swimming in extinct taxa. In addition, this finding has wider implications. It calls into question some previous paleotemperature estimates based partially on these taxa, suggests that the existing hypothesis about the evolution of regional endothermy in fishes requires modification, and provides key evidence for understanding the evolution of gigantism in active macropredators.

## Introduction

Otodontids or megatooth sharks are an extinct family of lamniform apex predators that lived from the Early Paleocene to Pliocene [1]. This group is only known from disarticulated remains, mainly isolated teeth but also some sets of associated teeth and vertebral centra [2–5]. The large size of these remains, including teeth that reach up to 168 mm in height [6], has aroused the interest in the group for some time. In consequence, several authors have tried to establish accurate allometric relationships between tooth height (or vertebral centra width) and body length and between body length and body mass in extant sharks with the goal of inferring sizes and masses of otodontids [6–8]. The most conservative estimates for body length of the popularly known “Megalodon” (*Otodus (Megaselachus) megalodon* following Capetta [1]) are about 16 meters, which makes it the largest megapredatory shark known among both fossil and extant taxa [6]. However, the scarcity of studies dealing with the factors that allowed megatooth sharks to reach such large sizes is striking. In this sense, some works (e.g., [6,9,10]) suggest that the gigantism as well as the distribution patterns, evolution and extinction of otodontids were closely linked to climatic factors and/or the abundance of their potential prey after the radiation of the cetaceans in the Paleogene.

Most of these previous hypotheses, however, presupposed otodontids as ectothermic fishes without taking into account some possible size-related metabolic constraints on the macropredatory lifestyle [11,12]. In this sense, there is a clear tendency for the largest marine vertebrates to be slow filter-feeding planktivores, whereas active macropredators are much smaller, suggesting that some size-related factors determine their activity and feeding strategy [13]. Most of the biggest ectothermic fishes, such as the whale shark (*Rhincodon typus*), the basking shark (*Cetorhinus maximus*) and the megamouth shark (*Megachasma pelagios*), are in fact filter-feeding chondrichthyans that range in size from 5.5 to 21 meters [14]. Strikingly, the only marine vertebrates with sizes and lifestyles comparable to those of the biggest megatooth sharks are large odontocete cetaceans with well-developed homeothermy, such as the killer whale (*Orcinus orca*) and the sperm whale (*Physeter catodon*) [15].

Contrary to all classical interpretations, Ehret [5] questioned the ectothermic nature of otodontids and suggested possible endothermy based on the lack of a correlation between their growth rates and the sea temperature through the Cenozoic, which would be expected if they were ectotherms. More recently, Pimiento et al. [16] have also proposed regional endothermy for *O*. *megalodon* on the basis of paleobiogeographical data and the range of water temperatures this species inhabited. Regional endothermy is the ability of some fish lineages to maintain certain body areas at higher temperatures than the surrounding water by means of vascular countercurrent heat exchangers or specialized thermogenic organs [17,18]. This adaptation involves an active mode of life and much higher metabolic rates than those of the ectothermic fishes of the same size [19,20]. Within osteichthyans, it has appeared independently in at least three different groups including tunas (tribe Thunnini, Scombridae), billfishes (Xiphiidae and Istiophoridae) and *Gasterochisma* [17]. Recently, whole-body endothermy has also been described in the opah (*Lampris guttatus*) [21]. In chondrichthyans, regional endothermy is restricted to lamniform sharks, where it is found in two of the three species of alopids [22–24] and in all species of the family Lamnidae ([25] and references therein).

Interestingly, regional endothermy of otodontids is rather parsimonious considering that they seem to be phylogenetically closely related to lamnids [5], according to two hypotheses about the relationships between the groups. The first considers otodontids as lamnids [6,9,26–29], while the second considers them as a separate family, which is either sister-group, or closely related to laminids [1,5,30–36]. In fact, some works have suggested that both groups could have evolved from representatives of other Late Cretaceous-Paleocene lamniform sharks, traditionally included in the family Cretoxhirinidae [1,9,26,31] (see Results and discussion section for a more detailed revision of the phylogenetic affinities of cretoxyrhinids).

Here it is proposed that regional endothermy was present in otodontids and some close related taxa (cretoxyrhinids), playing a key role in their evolutionary history, allowing gigantism and the maintenance of active macropredatory modes of life. Three approaches provide evidence for regional endothermy in these groups: (1) testing the degree to which their body temperature is dependent on that of the surrounding water by analysing δ^18^O differences between teeth of cretoxyrhinids-otodontids and associated ectotherms; (2) assessing whether burst swimming speeds of cretoxyrhinids-otodontids, estimated assuming ectothermy and regional endothermy, were fast enough for hunting successfully on their potential prey; and (3) assessing the energetic viability of ectothermy in cretoxyrhinids by calculating whether their costs of swimming were long-term sustainable by their metabolic budget. In addition, a number of multidisciplinary implications derived from this finding are discussed. These include: (1) the possible overestimation of some previous paleotemperature estimates based partially on these taxa, (2) the reinterpretation of some pre-existing hypotheses about the evolution of regional endothermy in fishes, and (3) the contribution of this work to the understanding of the evolution of gigantism in active macropredators.

## Data source and methodology

### Oxygen isotopic approach

Vertebrate oxygen isotopic data have been compiled from the literature including records from 20 different sedimentary beds, ranging in age from the Cretaceous to the Miocene, where remains of Cretoxyrhinidae and Otodontidae have been recovered in association with presumed ectothermic taxa (Table 1 and S1 Table). Following the methodology established by Bernard et al. [37], the difference in δ^18^O values between coexisting cretoxyrhinids/otodontids and ectothermic taxa has been calculated in all beds, plotting them as a function of the ectothermic taxa δ^18^O value (as a proxy of seawater temperature) and adjusting all points to a regression line whose slope is an indicator of the degree of thermoregulation. Slope values close to -1 imply that body temperature is independent of that of the water, slope values close to 0 imply a complete dependence, and intermediate slope values indicate some degree of independence. Three different regression analyses were performed: (1) including all cretoxyrhinids-otodontids and all associated ectothermic taxa; (2) including all cretoxyrhinids-otodontids and only associated pelagic ectothermic taxa (minimizing variability caused by the thermal gradient of the water column); and (3) including only cretoxyrhinids-otodontids and associated pelagic ectothermic taxa from Kocsis et al. [38], where remains considered as contemporary come from well delimited individual layers (reducing variability associated with low stratigraphic and temporal precision). Data from specimens doubtfully assigned to *Cretolamna* and *Otodus* or specimens where diagenetic alterations have been demonstrated were not included in the analyses. In order to avoid the inclusion of endothermic taxa as a proxy of water temperature, the following cases were also discarded: (1) data from expected endothermic taxa, such as mammals, ichthyosaurs, plesiosaurs, mosasaurs or some fish groups (see Introduction) and (2) data from chondrichthyans whose taxonomic assignment does not rule out the possibility of pertaining to one of the regional endothermic families.

**Table 1:** Mean δ^18^O values of cretoxyrhinids-otodontids and associated ectotherms from different sedimentary beds.

Code	Locality	Age	Cretoxyrhinid or otodontid taxa	Cretoxyrhinid or otodontid δ^18^O			Ectotherm δ^18^O			Ectotherm δ^18^O (without bentic genera)			Source
				n	δ^18^O	SD	n	δ^18^O	SD	n	δ^18^O	SD	
1	Cerro la Bruja, Peru	Miocene	*Otodus megalodon*	1	20.90		2	21.00	0.42	2	21.00	0.42	[39]
2	Sidi Daoui, Morocco	Upper Maastrichtian	*Cretolamna biauriculata marocana*	1	19.98		1	20.15					[40]
3	Oued Erguita, Morocco	Lower Maastrichtian	*Cretolamna*	1	18.95		1	18.66					[40]
4	Ouled Abdoun, Morocco	Upper Maastrichtian	*Cretolamna*	2	20.35	0.92	1	19.50		1	19.50		[37]
5	Ouled Abdoun, Morocco	Thanetian	*Cretolamna* sp.	1	19.47		1	19.98					[38]
6	Ouled Abdoun, Morocco	Thanetian	*Otodus* sp.	2	20.92	0.08	3	21.65	0.61	3	21.65	0.61	[38]
7	Ouled Abdoun, Morocco	Thanetian	*Otodus* sp.	2	20.49	0.04	1	20.57		1	20.57		[38]
8	Ouled Abdoun, Morocco	Maastrichtian	*Cretolamna marocana*	1	20.56		1	19.87		1	19.87		[38]
9	Ganntour Basin, Morocco	Maastrichtian	*Cretolamna marocana*	1	21.04		1	21.19		1	21.19		[38]
10	Ganntour Basin, Morocco	Maastrichtian	*Cretolamna marocana*	2	20.67	0.48	1	21.03		1	21.03		[38]
11	Ganntour Basin, Morocco	Maastrichtian	*Cretolamna marocana*	2	20.44	0.65	2	20.53	0.35	2	20.53	0.35	[38]
12	Ganntour Basin, Morocco	Maastrichtian	*Cretolamna marocana*	2	20.41	0.65	2	20.77	0.11	2	20.77	0.11	[38]
13	Ganntour Basin, Morocco	Maastrichtian	*Cretolamna marocana*	2	20.26	0.22	1	19.62		1	19.62		[38]
14	Ganntour Basin, Morocco	Maastrichtian	*Cretolamna marocana*	2	20.25	0.10	2	20.39	0.37	2	20.39	0.37	[38]
15	Ganntour Basin, Morocco	Maastrichtian	*Cretolamna marocana*	2	20.76	0.18	4	20.94	0.23	4	20.94	0.23	[38]
16	Yonne, France	Upper Campanian	*Cretolamna appendiculata*	1	21.00		1	21.10		1	21.10		[41]
17	Ardèche, France	Upper Aptian	*Otodus sp.	1	20.10		1	20.70					[41]
18	Skotniki, Poland	Lower Cenomanian	*Otodus appendiculatus	1	20.52		2	19.35	0.77	2	19.35	0.77	[42]
19	Asen, Sweden	Uppermost Lower Campanian	*Cretolamna appendiculata*	1	20.94		1	19.62		1	19.62		[42]
20	Benguerir, Moroco	Maastrichtian	*Cretolamna maroccana*	6	19.43	0.31	5	19.98	0.51	5	19.98	0.51	[42]

* *Cretolamna* specimens

In parallel, measurements of internal body organs/tissues and surrounding water temperature have also been compiled for three extant species of regional endothermic sharks from Lowe and Goldman [25] (S2 Table). Data were represented in a similar way that was done for isotopic data, calculating the differences in temperature between each pair of measurements for all sharks, plotting them as a function of the seawater temperature and adjusting all points to a regression line. Slopes were then contrasted with those obtained for δ^18^O values of cretoxyrhinids and otodontids in order to evaluate if the degree of thermoregulation is comparable between living and extinct groups.

Finally, latitudinal changes in δ^18^O tooth values of cretoxyrhinids and presumed ectothermic fishes were also compared from Campanian-Maastrichtian data compiled in Pucéat et al. [42] (S3 Table). δ^18^O values were represented as a function of latitude, regression lines were obtained for both groups and ANCOVA analysis was performed by means of PASW software. The same analyses were carried out considering the sea surface temperatures predicted from the δ^18^O values (S3 Table). Data from undetermined teeth were not included in either analysis.

### Cruise and burst speed inferences

Records of cruise and burst relative swimming speeds of living fishes have been compiled from the literature including both ectothermic and regional endothermic species, trying to cover a wide range of sizes and taxonomic groups (S4 Table). Data were log-transformed and plotted as a function of total fish length. Then, the scaling of both swimming speeds was studied in ectotherms and regional endotherms by linear regression analysis and differences between both groups were tested by ANCOVA analysis using PASW software. Cruise and burst swimming speeds of some cretoxyrhinids (*Cretolamna* and *Cretoxyrhina*) and otodontids (*Megalolamna*, *Otodus (Megaselacus)*, *Otodus (Otodus)* and *Parotodus*) were estimated from their total body lengths by interpolation of the regression analyses. Estimates were carried out considering them both as ectotherms and regional endotherms and 95% individual prediction intervals were calculated for all swimming speeds. Total body lengths of 17.9 m, 9.2 m, 6.8 m, 5.0 m, 6.4 m and 3.0 m have been considered here for *Otodus (M*.*)*, *Otodus (O*.*)*, *Parotodus*, *Megalolamna*, *Cretoxyrhina* and *Cretolamna* respectively. Body length estimations of cretoxyrhinids (*Cretoxyrhina* and *Cretolamna*) were taken from previous studies based on articulated or semi-articulated specimens [43,44]; whereas those of otodontids (*Megalolamna*, *Parotodus*, *Otodus (O*.*)* and *Otodus (M*.*)*) were based on analysis of isolated teeth figured in the literature. Following the methods described in Pimiento et al. [45], once a range of plausible positions was assigned to each tooth, crown height was used to calculate body lengths from the position-specific regressions established in white sharks by Shimada [8] (Table 2). Only one tooth per species was selected representing the largest or one of the largest records for its position in the dental series. This allows a conservative estimate of the body sizes of these taxa to be obtained, but larger individuals probably existed. In any case, determining the maximum body lengths of these sharks is beyond the scope of this paper and considering larger body size estimates would only favour the endothermic scenario (see Results and discussion section).

**Table 2:** Tooth crown heights (CH) and total body length (TL) estimates of otodontids and cretoxyrhinids. Tooth positions: A, upper anterior tooth; a, lower anterior tooth; L, upper lateral tooth.

Taxa	Source	CH (mm)	Tooth position	TL (m)
*Otodus (M*.*)*	[46]: Suppl. Mat. (UF 257579)	41.2	L5-L7	17.90*
*Otodus (O*.*)*	[1]: fig. 208A	65.0	a1-a2	9.18*
*Parotodus*	[1]: fig. 211A	48.0	a1-a2	6.76*
*Megalolamna*	[47]: fig. 2L	38.8	A1-A2 or a1-a2	5.02*
*Cretoxyrhina*	[43]	-	-	6.40
*Cretolamna*	[44]	-	-	3.00

* Total length estimates calculated from the average of length estimates for the different positions where each tooth could have belonged.

Independent estimations of cruise and burst swimming speed of *Cretoxyrhina* were obtained following the models developed by Sambilay [48] on the basis of the relationship between swimming speed and caudal fin aspect ratio:

$${\text{Log}}_{10}\left({\text{S}}_{\text{c}}\right)=-0.828+0.6196*{\text{Log}}_{10}(\text{PCL})+0.3478*{\text{Log}}_{10}(\text{AR})$$

$${\text{Log}}_{10}\left({\text{S}}_{\text{b}}\right)=-0.0659+0.6196*{\text{Log}}_{10}(\text{PCL})+0.3478*{\text{Log}}_{10}(\text{AR})$$

Considering AR = H^2^ / S

Where S_c_ is cruising swimming speed in kilometres * hours^-1^, S_b_ is burst swimming speed in kilometres * hours^-1^, PCL is precaudal body length (i.e., body length excluding the caudal fin or standard length) in centimetres, AR is aspect ratio, H is height of the caudal fin, and S is surface area of the caudal fin (note that H^2^ and S should be expressed in the same units because AR is dimensionless).

PCL of *Cretoxyrhina* was calculated assuming 640 cm TL from Mollet and Cailliet’s [49] equation:

PCL=−0.09195+0.8535*TL

The aspect ratio of *Cretoxyrhina mantelli* was inferred from two different caudal fin variables (Cobb’s angle and hypochordal ray angle, S1A Fig) measured on a well-preserved specimen (CMN 40906, S1B Fig). The relationship between such metric variables and AR was previously established in living lamniform sharks by linear regression from data provided in Kim et al. [50] (see S5 Table).

### Cost of swimming and energy budget inferences

The energy budget of *Cretoxyrhina mantelli* (assessed from its routine metabolic rate) was compared with independent inferences of its locomotion energy requirements (i.e., net cost of swimming), considering different water temperatures and both ectothermy and regional endothermy. The potential habitable temperature range (i.e., temperatures at which energy budget exceeds locomotion requirements) was then evaluated for each thermophysiological strategy and compared with pre-existent paleoclimatic data and the paleobiogeographic distribution of this shark. This allowed the plausibility of the ectothermic and the endothermic scenarios to be contrasted.

#### Net cost of swimming

Power-performance curves establish the relationship between relative swimming speed (U, body lengths*s^-1^) and oxygen consumption (MO_2_, mgO_2_*kg^-1^*s^-1^). Net costs of swimming at cruising speed (NCS) were calculated in *Cretoxyrhina* from power-performance curves of living sharks as the difference between the total metabolic rate (TMR, oxygen consumption at a particular swimming speed) and the standard metabolic rate (SMR, oxygen consumption at resting) (S2 Fig). Estimates were obtained from the equations established in *Carcharhinus acronotus* [51] (S2A Fig) and *Isurus oxyrinchus* [52] (S2B Fig), considered as ectothermic and regional endothermic oxygen consumption models respectively (see S1 Text for a detailed justification of this selection):

$${\text{Log}({\text{MO}}_{2})}_{C.acronotus}=2.38+0.377\text{U}$$

from which it is deduced that

TMR(U>0)C.acronotus=10^(2.38+0.377U)

SMR(U=0)C.acronotus=10^2.38

$$\text{Log}{\left({\text{MO}}_{2}\right)}_{I.oxyrinchus}=2.0937+0.97\text{U}$$

from which it is deduced that

TMR(U>0)I.oxyrinchus=10^(2.0937+0.97U)

SMR(U=0)I.oxyrinchus=10^2.0937

Where MO_2_ is oxygen consumption in mgO_2_ * kg^-1^ * s^-1^, U is relative swimming speed in body lengths * s^-1^, TMR is total metabolic rate in mgO_2_ * kg^-1^ * s^-1^, and SMR is standard metabolic rate in mgO_2_*kg^-1^*s^-1^.

Thus, NCS of *Cretoxyrhina* at cruising speed assuming ectothermy and regional endothermy was calculated as:

$${\text{NCS}}_{\text{Ectothermic}\;Cretoxyrhina}={\text{TMR}}_{\text{Ectothermic}\;Cretoxyrhina}-{\text{SMR}}_{\text{Ectothermic}\;Cretoxyrhina}$$

NCSEctothermicCretoxyrhina=[10^(2.38+0.377Uc)]−(10^2.38)

$${\text{NCS}}_{\text{R}.\;\text{endothermic}\;Cretoxyrhina}={\text{TMR}}_{\text{R}.\;\text{endothermic}\;Cretoxyrhina}–{\text{SMR}}_{\text{R}.\;\text{endothermic}\;Cretoxyrhina}$$

NCSR.endothermicCretoxyrhina=[10^(2.0937+0.97Uc)]−(10^2.0937)

Where NCS is net cost of swimming in mgO_2_ * kg^-1^ * s^-1^, TMR is total metabolic rate in mgO_2_ * kg^-1^ * s^-1^, SMR is standard metabolic rate in mgO_2_ * kg^-1^ * s^-1^, and U_c_ is relative cruising swimming speed in body lengths * s^-1^ calculated from [48]’s model, see above.

The use of NCS was preferred here instead of TMR as they are considered a better parameter for assessing the energy expenditure of thrust generation during swimming [53] and seem to be independent of the water temperature [54–56].

#### Routine metabolic rate

Routine metabolic rate (RMR) is the mean metabolic rate measured in an animal that performs random physical activity over a given period [57]. Here, the scaling of RMR with body mass has been independently established in ectothermic and regional endothermic fishes and used to infer the RMR of *Cretoxyrhina* assuming both conditions. For this, 43 records of RMR of extant sharks and rays were compiled from the literature (S6 Table). RMR data were temperature adjusted to 5°C, 10°C, 15°C, 20°C, and 25°C using Q_10_ of 2.3 (following [19]). RMR and body mass data were log-transformed and regression lines were fitted independently for points corresponding to ectothermic and regional endothermic species at each temperature. The RMR of *Cretoxhirina* was then inferred in all possible scenarios, assuming a body mass of 2655 kg (calculated from the exponential model proposed by Gottfried et al. [6] assuming a total body length of 6.4 m [43]).

#### Validation Test

Following the same procedure, the thermophysiological strategy of 17 living sharks, including 14 ectothermic species and 3 regional endothermic species, was assessed from simultaneous records of their cruise swimming speeds, water temperatures and body masses (S7 Table).

## Results and discussion

### Evidence of regional endothermy in otodontids and cretoxyrhinids

#### Oxygen isotopic approach

The oxygen isotopic composition of bioapatite phosphate and carbonate has been extensively studied in vertebrates for different purposes (see [58] for a detailed review). Given that δ^18^O value of a biomineral depends both on the δ^18^O value of the body fluid from which it precipitates and the temperature at which it forms [58], some authors have used oxygen isotopic data from bone, dentine or enamel to infer high body temperatures or thermoregulation in fossil taxa (e.g., [37,59–62]). Recently, Bernard et al. [37] have established a useful approach for inferring the dependence of body temperature of extinct aquatic animals on that of the surrounding water (see Data source and methodology section), demonstrating thermoregulation in some Mesozoic marine reptiles. Following this methodology, the thermophysiology of cretoxyrhinids and otodontids has been explored here from the isotopic composition of fossil remains from 20 different sedimentary beds, ranging in age from the Cretaceous to the Miocene (Table 1 and S1 Table).

**Fig 1:**
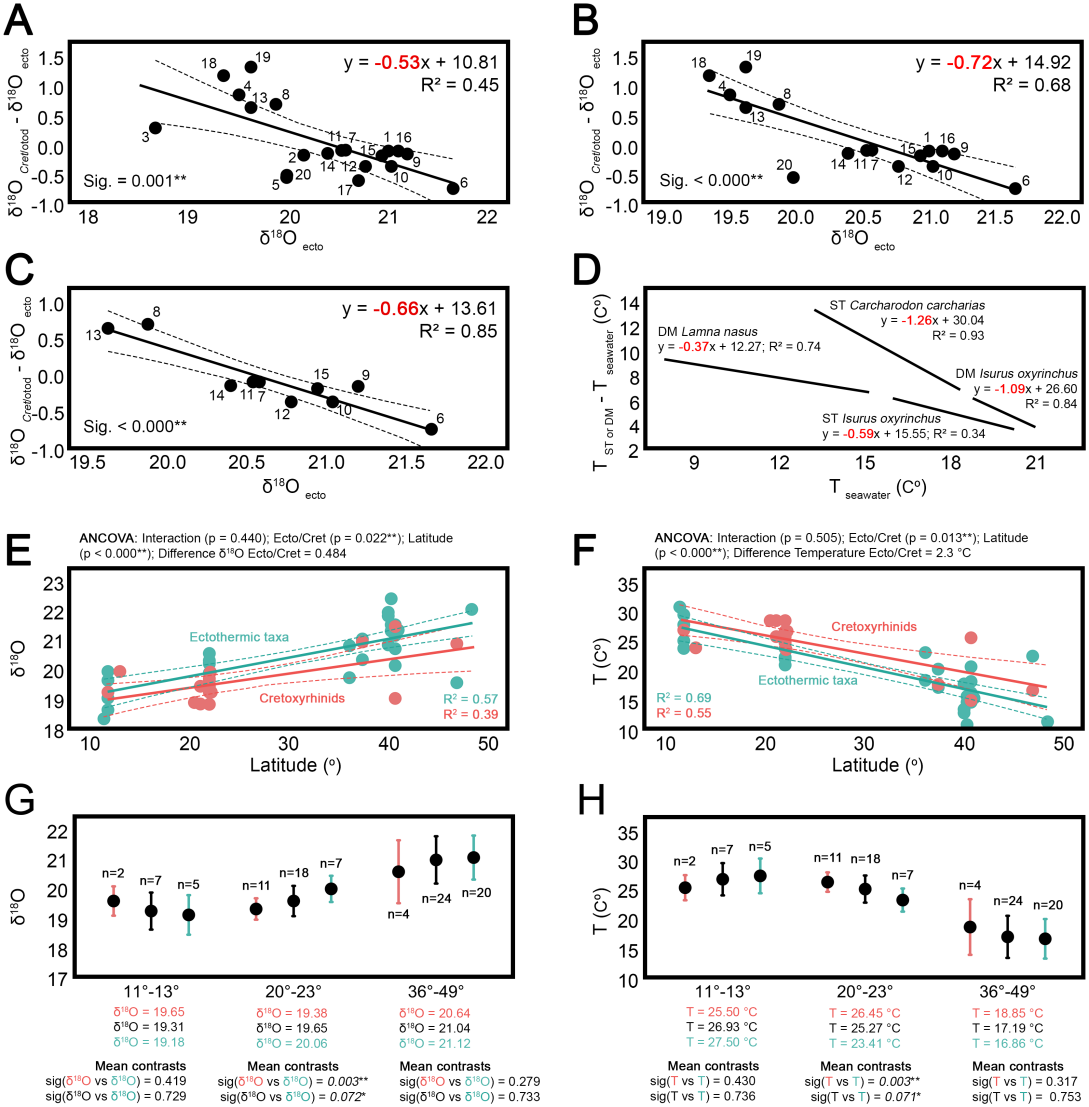
Oxygen isotopic evidence and the effect of regional endothermic taxa in previous paleotemperature estimates. (A-C) Difference in the δ^18^O value between coexisting cretoxyrhinids/otodontids and ectothermic taxa in several localities/sedimentary beds plotted against the ectothermic taxa δ^18^O value (as a proxy of seawater temperature). Three different regression analyses have been performed: (A) including all cretoxyrhinids-otodontids and all associated ectothermic taxa, (B) including all cretoxyrhinids-otodontids and only associated pelagic ectothermic taxa; and (C) including only cretoxyrhinids-otodontids and associated pelagic ectothermic taxa from Kocsis et al. [38] (regression lines are showed with associated 95% confidence intervals). Details of each fossil locality (denoted by numbers) are given in Table 1 and S1 Table. (D) Regression analyses performed in living lamnid sharks for comparative purposes, considering direct water and body temperature records compiled in Lowe and Goldman [25] (DM, deep muscle; ST, stomach). In all cases, slope values close to -1 imply that body temperature is independent from that of the water, slope values close to 0 imply a complete dependence, and intermediate slope values indicate some degree of independence. (E-F) Campanian-Maastrichtian latitudinal gradients of δ^18^O and seawater temperature calculated from cretoxyrhinids and ectothermic taxa. (G-H) Campanian-Maastrichtian δ^18^O and seawater temperature estimates calculated for three different latitudinal ranges (11°-13°, 20°-23° and 36°-49°), considering only cretoxyrhinids (pink), all taxa (black) and only ectothermic taxa (green). Significance of pairwise mean contrasts are shown in each case. Data in E-H taken from Puceat et al. [42]. ** indicates significance at the 0.05 level and * indicates significance at the 0.1 level.

The regression line slopes obtained for the analysis of *Cretolamna*-*Otodus* species are significantly different from zero and their values are consistent with the existence of partial independence from sea water temperature (Fig 1A–1C). These results are comparable in some aspects with those provided by Bernard et al. [37], showing similar fits in the regression lines and similar standard deviation values for isotopic data (see Table 1 and compare with [37]: table S1). Furthermore, a significantly higher number of localities has been considered here, increasing the reliability of the regression results and allowing consistent conclusions. In addition, some considerations have been taken into account in order to remove or minimize possible biases related to preservation or ecological aspects. Firstly, although diagenesis may occur in a biomineral altering the δ^18^O composition, enameloid is rather resistant to postdepositional alteration and recrystallization due to its dense and slightly porous structure [58]. Accordingly, almost all δ^18^O data selected here come from measurements taken in tooth enameloid (except for two turtle osteocutes and a few taxa where the whole tooth was considered, S1 Table). In fact, there are previous Rare Earth Elements analyses [40,63–65] as well as taphonomic evidence [38,39] for almost all the teeth included in this analysis supporting the lack of diagenetic processes (see [66] for a detailed review on the applicability of REE analysis as a tool for detecting diagenetic processes). Moreover, Žigaitė and Whitehouse [67] have recently studied the isotopic composition of different tooth tissues in extant sharks demonstrating that enameloid shows less δ^18^O variation than dentine and recommended it as a target biomineral and as a preferential biogeochemical reference for environmental and paleoenvironmental studies (see also [68]). Secondly, δ^18^O differences between teeth of co-occurring individuals can also reflect differences in water column temperature due to distinct water depth preferences [40,69,70]. In order to minimize this bias, isotopic data from benthic ectothermic vertebrates were discarded from adjusted regression lines, leaving only taxa with similar ecology to cretoxyrhinids and otodontids as water temperature proxies. As expected, the effect of water column temperature variations became evident when a wide ecological range of taxa are considered, resulting in less well fitting regression lines including benthic taxa (R^2^ = 0.45, Fig 1A) than excluding them (R^2^ = 0.68, Fig 1B). Finally, individuals collected from the same sedimentary bed may not be strictly contemporaneous and differences in δ^18^O values could be reflecting seasonality or climate change. Although this problem can never be ruled out, accurate stratigraphic knowledge of layers that yield the presupposed contemporaneous remains can minimize it. In this sense, the best regression line fit is obtained taking into account only taxa included in Kocsis et al. [38] (R^2^ = 0.85, Fig 1C), where the layer of provenance of each specimen is specified accurately (note that the stratigraphic resolution of some of the other localities is given only at bed level, S1 Table).

The existence of some decoupling between the enamel δ^18^O composition of cretoxyrhinids-otodontids and coetaneous ectothermic organisms implies the presence of more constant body temperatures in the head and jaws of the former. Body temperature decreases towards the exterior in extant regional endotherms and is almost equal to the surrounding water temperature on the body surface [71–74]. Thus, the influence of the changes of water temperature it is expected to be more noticeable on external body parts, such as the region where teeth calcify. Unfortunately, this influence has never been quantified in this area and direct comparisons with results obtained for cretoxyrhinids and otodontids are not possible. However, elevation in deep muscle and stomach temperature over ambient temperatures has been recorded in some species of extant regional endothermic sharks [25]. Here, the influence of water temperature on the deep muscle of *Isurus* and *Lamna* and on the stomach of *Isurus* and *Carcharodon* has been quantified from measurements reported in previous works (S2 Table). The representation of these temperature data in the same way as the isotopic data allows a comparison between the slopes obtained in both cases given that δ^18^O values reflect water and body temperatures indirectly (Fig 1D). The influence of surrounding water temperature on teeth in formation of cretoxyrhinids and otodontids (slope value of -0.66, Fig 1C) seems comparable to that on the stomach of *Isurus* and less important than on the muscle of *Lamna* (slope values of -0.59 and -0.37 respectively; Fig 1D). In any case, although calcifying teeth are embedded in relatively superficial tissues, this result might be expected as many regional endonthermic fishes have cranial endothermy. Namely, lamnids, xiphids, istiophorids and some tunas are able to elevate the temperature of their brains and nearby structures ([75] and references therein). In lamnids this is achieved mainly by means of a red muscle vein that transfers heat from red muscles to brain and orbital retia, but also by the heat supplied by some aerobic muscles, such the eye muscles, and other metabolically active tissues [76]. Interestingly, Tubbesing and Block [76] noted that jaw muscle of *Lamna nasus*, composed of slow-twitch aerobic fibbers, could also aid in local heat generation, being a closer possible heat source for dental lamina. In sum, the presence of different mechanisms that contribute to tissue warming in the head of some lamniform sharks makes the existence of high temperatures in the area where teeth calcify likely, affecting the isotopic composition of enameloid in developing teeth. In fact, the effect of cranial endothermy on the isotopic composition of other calcified structures of the head has already been demonstrated in tuna otoliths [77]. However, studies focused on recording temperatures in dental lamina of the upper and lower jaw in regional endotherms are needed in order to determine the degree of temperature independence of these tissues and the possible influence of cranial endonthermy on the oxygen isotopic composition of teeth.

#### Cruise and burst swimming speed inferences

**Fig 2:**
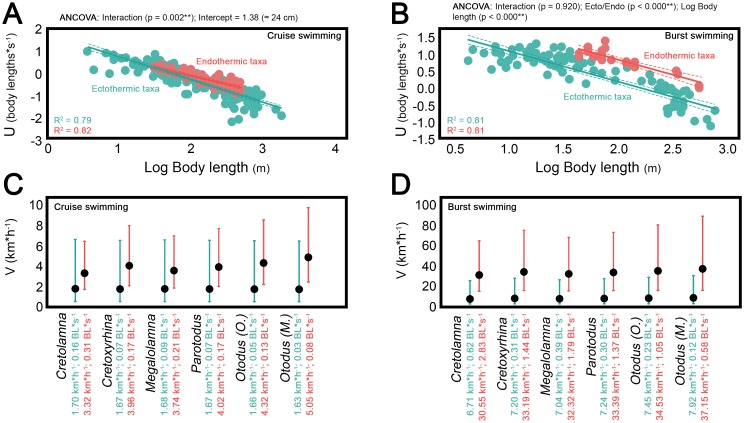
Scaling of swimming speed in extant fishes and swimming speed inferences in cretoxyrhinids and otodontids. (A) Cruise and (B) burst relative swimming speeds (U, body lengths*s^-1^) against body lengths (meters) of living ectothermic and regional endothermic fishes. Adjusted regression lines are showed with associated 95% confidence intervals. (C) Cruise and (D) burst swimming speed estimates (V, km*h^-1^) of cretoxyrhinids and otodontids, considering them as ectothermic sharks (green) or regional endothermic sharks (pink), with associated 95% individual prediction intervals. Values of absolute (V, km*h^-1^) and relative (U, BL*s^-1^) speed estimates are also shown for each case. ** indicates significance at the 0.05 level.

The scaling of fish locomotion has been comprehensively studied both in terms of steady and unsteady swimming, and it is well known that cruise and burst relative speeds decrease with body length [78–82]. The reasons underlying this phenomenon are related to physiological constraints that imply lower muscle contraction rate when body mass increases [83,84], resulting in lower tail beat frequencies in larger fishes [79]. However, although the scaling of locomotion is well known for fishes in general, there are virtually no works comparing the relationship between swimming speed and body size in ectothermic and regional endothermic taxa (with the only exception of [85]). Here, fish swimming speed data have been compiled for extant species covering a wide range of body sizes (S4 Table) and this relationship has been studied and compared in both groups. ANCOVA analysis, adjusted regression lines and their associated confidence intervals strongly support the existence of higher cruise and burst swimming capabilities in regional endotherms (Fig 2A and 2B).

Some works have previously suggested differences in swimming speeds of regional endotherms and ectotherms on the basis of indirect evidence such as the increase in power output and muscular contraction rate at high body temperatures, the enhancing of the diffusion of oxygen to the muscle mitochondria and increasing lactate turnover ([18] and references therein). Nonetheless, direct records of swimming speeds are scarce and sometimes inconclusive given the size limitations of controlled experiments and the difficulty of taking measurements in the wild. Regarding cruising speeds, some experiments carried out in water tunnels suggested similar speeds for ectothermic and regional endothermic taxa [86–91]. However, the work of Watanabe et al. [85] has recently shed light on this, demonstrating that cruising speeds of fishes with regional endothermy are greater than fishes without it using free-swimming data. They argued that previous results obtained under non free-swimming conditions may be explained by the small size of water tunnels that do not allow the study of big adults where the regional endothermy is well developed. ANCOVA analysis performed here also suggest that differences in cruise swimming capabilities of ectothermic and regional endothermic fishes increase gradually with body size (Interaction, p = 0.002; Fig 2A). The intersection of adjusted lines occurs at 24 cm total length meaning that cruising speeds of both regional endotherms and ectotherms are equal at that size. Interestingly, Dickson [92] and Dickson et al. [93] have proposed similar lengths as minimum sizes of functional endothermy in fishes. A high surface area-to-volume ratio constrains the ability to maintain warm tissues in smaller individuals and endothermy cannot be completely developed until a threshold mass is reached [94]. On the other hand, works comparing burst swimming speeds of ectothermic and regional endothermic fishes from direct measurements are virtually non-existent. Some extraordinarily high speed records of small ectothermic sphyraenids and scombrids (9.5 BL*s^-1^ and 19.4 BL *s^-1^ for *Sphyraena barracuda* and *Acanthocybium solandri* respectively) equal or exceed the maximum speeds documented in regional endotherms of similar sizes (See S4 Table). These data and the methodology by which they were obtained have been sometimes questioned (see [95–99]). However, high burst speeds could be expected for these and other tropical or subtropical species inhabiting warm waters as the contraction time of the anaerobic muscles involved in burst swimming becomes shorter when temperature rises [100]. In any case, here, ANCOVA analysis detects differences between burst speeds of both groups even when these bizarre speeds are taken into account (Interaction, p = 0.966; Ecto/Endo, p < 0.000; data not shown in Fig 2B).

Scaling relationships of cruise and burst swimming speeds for both ectotherms and regional endotherms fit well into linear models (Fig 2A and 2B). Comparatively low dispersion of the data has made it possible to obtain narrow individual confidence intervals allowing swimming speed predictions in new taxa accurate enough for the purpose of the present study. Cruise and burst swimming speed estimates have been obtained for extinct cretoxyrhinids and otodontids from their total body lengths (Fig 2C and 2D). Previous estimates of swimming speeds of otodontids have only been provided by Jacoby et al. [101], predicting a cruising swimming speed for *Otodus (Megaselachus) megalodon* (1.34 m*s^-1^) very similar to the values obtained here assuming regional endothermy (1.40 m*s^-1^). On the other hand, there are no studies so far aiming to assess the burst swimming speeds of cretoxyrhinids and otodontids, this work being the first attempt in this sense. Unsteady swimming locomotion (i.e., burst speed) is extremely important in prey-predator interactions, mainly when prey/predator length ratio is high (similar to or higher than 10^−1^) and feeding strategy implies a whole body attack [102,103]. Otodontids and cretoxyrhunids are thought to have been active macropredators hunting on relatively big and fast swimming prey. This interpretation is based in functional analyses of their dentitions [1,31], trophic level inferences from isotopic data [104] and direct evidence such as coprolites with fish remains [105] or bite marks, fractures and embedded teeth in fossil cetacean, sirenian and marine reptile bones [6,9,10,106–115]. As a most extreme case, some of the largest representatives of the group were potential predators of big-sized marine mammals ([16] and references therein), possibly exerting an important control on their communities and even playing a significant role in the evolutionary history of big filter-feeding whales [116] (although see also [115]). Consequently, it seems obvious that reaching high swimming speeds should be also crucial for the hunting success of cretoxyrhinids and otodontids. In this sense, the range of burst swimming speeds inferred here considering them as ecthothemic sharks (6.7–7.9 km*h^-1^) seems to be too low for such active macropredators. This is especially drastic for the case of the biggest species because their absolute speeds correspond to extremely low relative speeds (e.g., 0.12 body lengths*s^-1^ in *O*. *megalodon*) and hunting success depends largely on the later [117]. In contrast, burst swimming speeds estimated considering both groups as regional endotherms (30.6–37.2 km*h^-1^) appear to fit better with their presupposed lifestyles, probably being high enough for active pursuit and hunting of fast prey (burst swimming speeds of extant big and medium-sized odontocetes, sirenians and otariids range approximately between 20 km*h^-1^ and 30 km*h^-1^ [118–121]).

In addition, the squamation pattern and some morphofunctional interpretations of cretoxyrhinids also support an adaptation to fast swimming, more congruent with values estimated for the scenario of regional endothermy. Shimada [122] noted that the body surface of *Cretoxyrhina* was covered by scales with parallel keels separated by U-shaped grooves where the average interkeel distance was approximately 45 microns (see [43]: fig 5 and [122]: fig 8B). These aspects evidence a clear role in drag reduction and allow the unequivocal assignation of *Cretoxyrhina* scales to a morphotype that is exclusive to the fastest living species of sharks [123–127]. In the same way, some other evident aspects of the squamation of *Cretoxyrhina*, such as the high density of scales together with a notable overlapping ([122]: fig. 8A and B) or the presence of scales with crown thinning ([43]: fig. 5A and C), have also been interpreted as an adaptation for enhancing hydrodynamic efficiency in fastest pelagic species ([128] and references therein). A few smooth rounded scales have also been described in *Cretoxyrhina* but they were probably restricted to the snout and possibly other regions exposed to high abrasive stress (scales of Type A in [122]). Similarly, functional interpretations of caudal fin remains also support the idea of *Cretoxyrhia* as a fast pelagic hunter. Specimen CMN 40906 exhibits the highest Cobb’s and hypochordal ray angles ever recorded in lamniform sharks (49° and 133° respectively), implying fast swimming capabilities in *Cretoxyrhina* [50]. Thomson and Simanek [129] noted that Cobb’s angles above 30° are characteristic of fast swimming pelagic sharks typified by the endothermic sharks within the family Lamnidae. In fact, the swimming speed calculated from morphological variables of the caudal fin of *Cretoxyrhina* (≈70 km*h^-1^, see S2 Text) is clearly incompatible with those predicted here under the assumption that it was an ectothermic shark (≈7 km*h^-1^). Finally, some other aspects, like the conical head and the shape and number of vertebra in *Cretoxyrhina* [122] and the absence of dorso-ventral flattening in *Cretolamna* vertebra [44], also point towards the existence of fusiform bodies in cretoxyrhinids compatible with an active pelagic lifestyle.

On the other hand, besides burst speed, some other unsteady swimming performance parameters, such as acceleration and manoeuvrability, are also size-constrained and decrease with body length [103]. For that reason, hunting strategies implying ambushing behaviour and/or attacks under specific ambient conditions could be reasonable for massive otodontids minimizing prey reaction time in a similar way to living great white sharks [130]. In fact, Godfrey and Altman [131], based on the analysis of a Miocene cetacean vertebra with a partially healed compression fracture, suggested that *O*. *megalodon* could display comparable predatory strategies to those of extant great white sharks. Similarly, predatory strategies implying group hunting are also effective when feeding on prey of similar or larger size than the predator [103]. However, although some modern marine vertebrates display examples of cooperative attacks (i.e., the killer whale *Orcinus orca*) (see for example [132]), this behavior has never been reported so far in big sharks and it is quite likely that *O*. *megalodon* was a solitary hunter [133]. In any case, any interpretation on the hunting strategies of large otodontids is highly speculative.

#### Costs of swimming and energy budget inferences

The comparison of locomotion costs with the metabolic ceilings of ectotherms and endotherms (i.e., energy budget) has been revealed as a useful approach for assessing the thermophysiological strategies of fossil groups [134]. Following a similar procedure, here the thermophysiology of *Cretoxyrhina mantelli* has been assessed by inferring and comparing estimates of its net costs of swimming and energy budget under ectothermic and regional endothermic scenarios.

**Fig 3:**
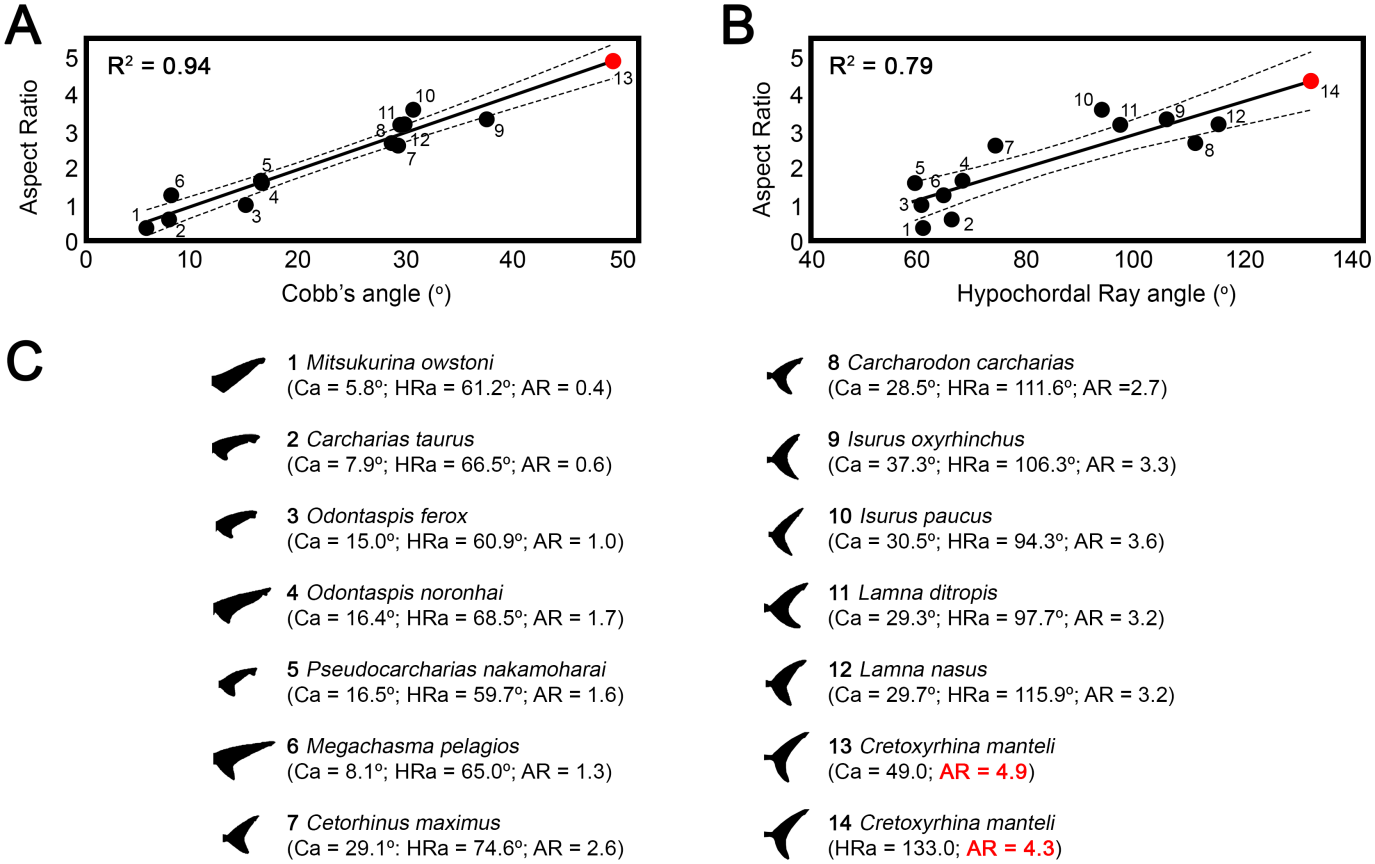
Aspect ratio estimates of *Cretoxyrhina mantelli*. (A-B) Regression analyses between the aspect ratio (AR) and two different caudal fin variables (Ca, Cobb’s angle; HRa, hypochordal ray angle) of living lamniform sharks. Extrapolated position of *Cretoxyrhina mantelli* is denoted by a red dot. (C) Morphometric data for caudal fins (AR, Ca and HRa) of lamniform sharks including *Cretoxyrhina mantelli*. Caudal fin profiles modified from Kim et al. [50].

Power-performance curves of continuously active elasmobranch species have been used by several authors for different purposes: (1) determining standard metabolic rates (SMR) extrapolating to 0 velocity (e.g., [51,135,136]) and (2) determining field total metabolic rates (TMR) at a given measured speed [137,138]. In the present study, for the first time, power-performance curves of living sharks have been used to estimate the locomotion energy requirements in an extinct taxon, *Cretoxyrhina mantelli*. A cruise swimming speed of 12 km*h^-1^ (0.53 body lengths*s^-1^) has been inferred here for this species after applying Sambilay’s [48] model assuming a total body length of 6.4 m and aspect ratio of 4.3 (See Fig 3 and S2 Text for further details of the aspect ratio and swimming speed inferences). At this speed, the net cost of swimming is 373 gO_2_*kg^-1^*h^-1^ and 622 gO_2_*kg^-1^*h^-1^ assuming ectothermy and regional endothermy respectively (Table 3). On the other hand, energetic budgets and metabolic ceilings in animals are usually assessed by calculating sustained metabolic rate (SusMR, metabolic level that can be sustained long-term by an animal) [139]. Unfortunately, there are virtually no works dealing with SusMR of sharks and the data available in the literature are insufficient for establishing predictive models. However, SusMR is considered as broadly equivalent in ecological terms to another much more frequently measured parameter, the routine metabolic rate (RMR) [140,141]. The well-fitted scaling relationships between the RMR and body mass obtained here for ectothermic and regional endothermic living sharks has allowed reliable predictions of RMR to be obtained in *Cretoxyrhina* (as an approximation of its metabolic budget) within narrow values of confidence (Table 3).

**Table 3:** Predicted values of net cost of swimming (NCS) and routine metabolic rate (RMR) in *Cretoxyrhina mantelli* assuming ectothermy and regional endothermy at five different scenarios of water temperature.

Predicted logNCS (mgO_2_*kg^-1^*h^-1^) _*Cretoxy*._	Regr. logRMR-logBody mass			
	Scenario		R^2^	Predicted logRMR (mgO_2_*kg^-1^*h^-1^) _*Cretoxy*._
5.572	Ectothermy	5°C	0.97	4.716 ± 0.344
5.572	Ectothermy	10°C	0.97	4.897 ± 0.344
5.572	Ectothermy	15°C	0.97	5.078 ± 0.344
5.572	Ectothermy	20°C	0.97	5.259 ± 0.344
5.572	Ectothermy	25°C	0.97	5.440 ± 0.344
5.794	R. endothermy	5°C	0.95	5.780 ± 0.486
5.794	R. endothermy	10°C	0.95	5.961 ± 0.486
5.794	R. endothermy	15°C	0.95	6.142 ± 0.486
5.794	R. endothermy	20°C	0.95	6.323 ± 0.486
5.794	R. endothermy	25°C	0.95	6.504 ± 0.486

**Fig 4:**
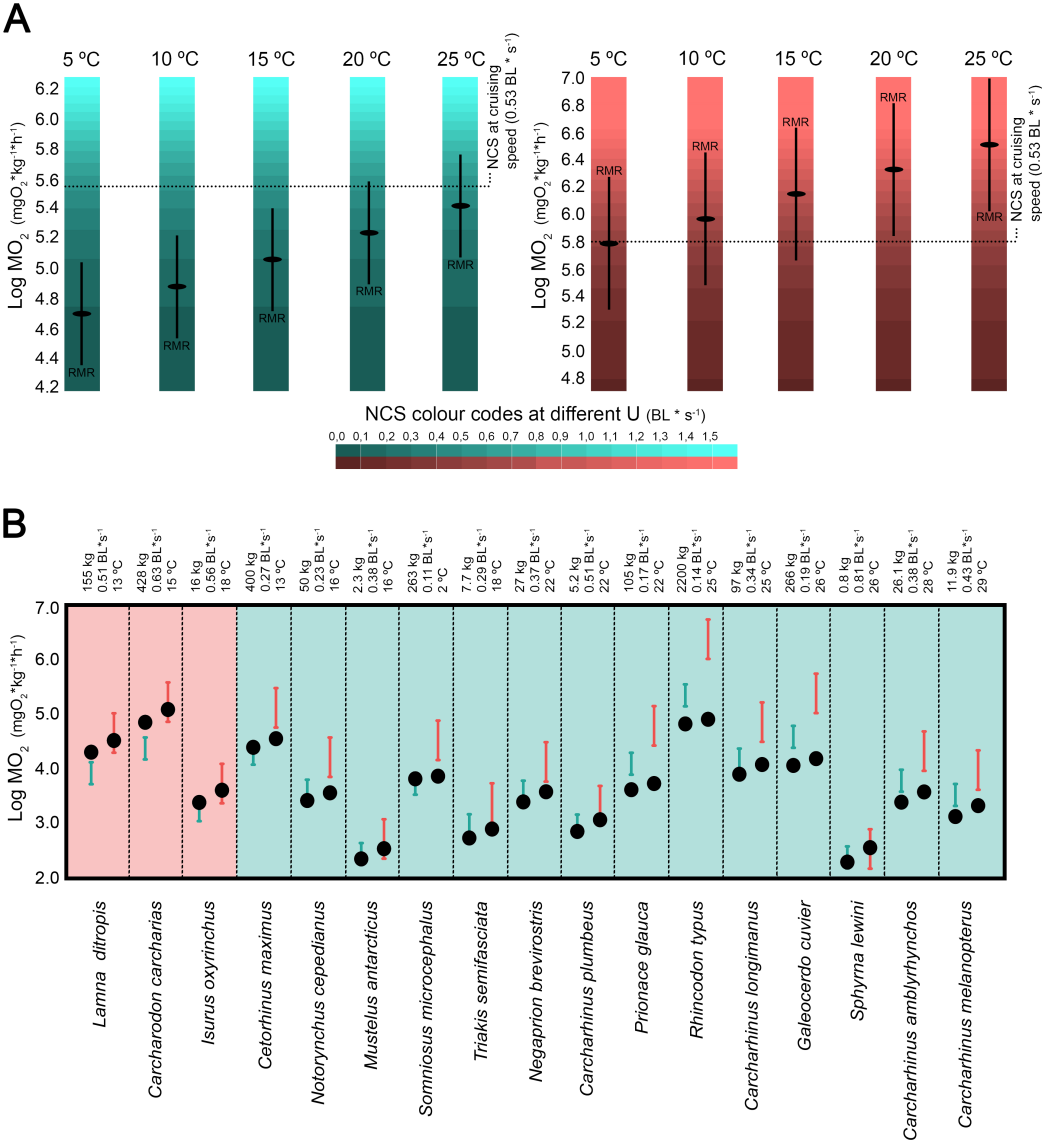
(A) Comparison between net cost of swimming (NCS) and routine metabolic rate (RMR) of *Cretoxyrhina mantelli* at five different temperature scenarios (5°C, 10°C, 15°C, 20°C and 25°C) assuming ectothermy (green) and regional endothermy (pink). Green and pink gradations represent the NCS at different swimming speeds (see color code chart). Note that NCS are constant in all temperature scenarios (see text). RMR estimate is represented with associated 95% individual prediction intervals (in black). (B) Validation test performed in 17 living sharks, including ectothermic taxa (green background) and regional endothermic taxa (pink background), from simultaneous records of their cruise swimming speeds, water temperatures and body masses (data taken from [85]). Inferred RMR are denoted as green and pink intervals for the ectothermic and regional endothermic scenario respectively; Inferred NCS are represented by black dots.

Metabolic ceilings have been proposed several times as constraining the distribution of plants and animals, accounting for their observed latitudinal and altitudinal limits [142–144]. Differences in thermal physiology between ectotherms and endotherms affect their global distributions differently, with ambient temperature being the main constrain for ectothermic organisms [145–147]. Accordingly, this work suggests differences in the potential habitable temperature range of *Cretoxyrhina* depending on its thermoregulatory capabilities. When considering *Cretoxyrhina* as an ectothermic shark the model predicts that its energy expenditure of locomotion would not be sustainable over time in waters below 20°C (note in Fig 4A that NCS exceeds RMR below this temperature in the ectothermic scenario). In contrast, such costs would be probably sustainable long-term by a regional endothermic shark in a wider range of water temperatures (RMR could exceed NCS at all the considered temperatures in the regional endothermic scenario, Fig 4A).

*Cretoxyrhina* remains have been documented from the Albian to the Cenomanian (Lower to Upper Cretaceous) over a period when sea surface mean temperatures were higher than nowadays and latitudinal gradients were less important [148]. Despite that, the fossil record of this shark reveals a cosmopolitan distribution with a paleolatitudinal range wide enough to suggest a broad tolerance to different water temperatures (see [1] for a summary of fossil occurrences). Paleobiogeographical data within the Western Interior Sea (USA) prove that *Cretoxyrhina* was able to inhabit conditions ranging from subtropical to cool temperate waters [149] and probably to migrate through the Boreal ocean [150]. In fact, the most northern fossil assemblages where *Cretoxyrhina* remains have been recovered demonstrate that this shark was, indeed, able to inhabit circumboreal seas where the mean annual surface temperature has been estimated to have been between 5°C and 10°C [151]. In consequence, the scenario of *Cretoxyrhina* as an ecothermic shark does not fit the paleobiogeographical and paleoclimatic evidence. The model presented here predicts that, in the absence of regional endothermy, only swimming speeds around or below to 0.1 body lengths*s^-1^ would be sustainable long-term for sharks of comparable body mass at 5°C (Fig 4A). Accordingly, very similar speeds have been reported for *Somniosus microcephalus* (0.11 body lengths*s^-1^ and 1.22 km*h^-1^) [152], the biggest ectothermic shark inhabiting polar waters [153]. On the contrary, the scenario that assumes regional endothermy seems to be more congruent with all these aspects. In that case, the temperature at which the NCS of *Cretoxyrhina* equates its RMR (≈5°C; Fig 4A) fits well within the lowest water temperatures inhabited by this taxon according to its northernmost distribution limit (5°C– 10°C according to [151]).

Interestingly, a validation test performed in 17 living sharks from simultaneous records of their cruise swimming speeds, water temperatures and body masses supports the notion that this approach has a high predictive power, as it is able to correctly predict the thermophysiological strategy of the vast majority of the species (Fig 4B). Energetic viability of ectothermy is supported for all the 14 ectotherm species studied with no exception (inferred NCS lay below or within the confidence intervals of the estimated RMRs). On the other hand, regional endothermy is predicted as the only energetically sustainable strategy in two regional endotherms, *Lamna ditropis* and *Carcharodon carcharias* (note that ectothermy cannot be rejected as a sustainable strategy in a third regional endothermic taxon, *Isurus oxyrhinchus*). Thus, the recognition of the ectothermic scenario as a non-energetically viable possibility is highly indicative of regional endothermy, this being the case for *Cretoxyrhina mantelli*.

### Implications of the regional endothermy of otodontids and cretoxyrhinids

Three independent lines of evidence (i.e., isotopic data, swimming speed estimates and metabolic inferences) strongly support the starting hypothesis of this work, strengthening the evidence for the existence of regional endothermy in otodontids and cretoxyrhinids. The detection of this adaptation in such extinct groups entails a series of implications, some of them with important multidisciplinary significance.

#### Implications for sea surface paleotemperature estimations

Several works have used the oxygen isotope composition of vertebrate remains for paleotemperature inferences of past seawater (e.g., [40–42,69,154–160] and many more). Most of them have been carried out by analyzing the enameloid from teeth and scales of fishes, without an exhaustive selection of the included taxa, assuming a unique fractionation equation applicable to all the species [41]. However, the presence of regional or whole endothermy in some fish lineages could affect the oxygen isotopic fractionation in their biominerals [58]. In fact, a number of studies have empirically demonstrated differences in the δ^18^O isotopic composition of mineralized tissues of coexisting taxa due presumably to differences in thermoregulatory physiology (e.g., [37,39,62]). In this sense, it is striking that several works aiming to reconstruct paleotemperatures of seawater [39,156,158] or interpret hydrographic changes [64] have ignored this fact, including δ^18^O isotopic data from taxa belonging to groups with regional endothermy, such as lamnids (e.g., *Carcharodon*, *Isurus*, *Isurolamna*, *Cosmopolitodus* and *Macrorhizodus*) or alopids (e.g., *Usakias*). Likewise, δ^18^O isotopic composition of teeth of otodontids and cretoxyrhinids has also been used for similar purposes (see for example [38–42,64,154–156,158,161]), without taking into account that these groups are potential regional endotherms given their close phylogenetic relationship with lamnids. The presence of cretoxyrhinids is especially frequent in works dealing with paleoenvironmental reconstructions of the Late Cretaceous-Paleocene as a result of the high abundance of *Cretolamna* teeth in sediments of that age. For instance, among the 108 fossil remains considered by Kocsis et al. [38], 30 are teeth belonging to *Cretolamna* and *Otodus*, constituting 28% of the total sample and up to 50% in some of the studied localities. Similarly, 24% of all teeth analyzed by Pucéat et al. [42] belong to cretoxyrhinids (*Cretolamna* and *Archaelomana*) and otodontids (*Otodus*). Among these works, the study carried out by Amiot et al. [39] is probably one of the most remarkable cases where only two (15%) of all included shark teeth belong unquestionably to ecothermic groups, whereas the remaining 11 teeth belong to otodontids (8%) and lamnids (77%). In addition, some of these studies include shark teeth with poor taxonomic determination meaning that it is not possible to rule out, in most cases, the possibility that they belong to one of the regional endothermic groups.

The evidence provided in the present work in favour of cretoxyrhinids and otodontids as regional endothermic sharks imply that some of the previous paleotemperature reconstructions based partially on these taxa could be overestimated and should be revised. As proof of this, the effect of including representatives of such groups in this kind of studies has been tested here considering the isotopic data provided by Pucéat et al. [42]. ANCOVA analyses support this prediction detecting significant differences both in the δ^18^O values and temperatures calculated from remains of cretoxyrhinds and ectothermic taxa at different latitudes (δ^18^O, p = 0.022; Temperature, p = 0.013; Fig 1E and 1F). Parameter estimation of ANCOVA analysis indicates that temperatures inferred from creotxyrhinids are 2.3°C above those predicted from ectothermic fishes (Fig 1F). These differences are expected to be more important in high latitudes where body temperature of regional endothermic animals is much higher than that of the surrounding water (see for example [162,163]). However, no significant differences have been found in the slope of ecothermic taxa and cretoxyrhinids (Interaction, p = 0.505; Fig 1F), possibly due to the small sample size of the latter in latitudes above 30°. In any case, the effect of cretoxyrhinids on previous sea paleotemperature reconstructions becomes clear when temperatures are re-estimated removing the isotopic data of such taxa from the analysis. Overestimations of up to 1.9°C have been calculated here for latitudes around 20° where a large number of *Cretolamna* teeth were taken into account (Note that no significant differences have been detected in latitudes around 10° and 40° probably due to the smaller sample size of cretoxyrhinids considered in those cases, Fig 1H). Hence, in view of these results, remains of regional endothermic taxa or closely related groups should be treated more cautiously in future studies dealing with seawater paleotemperature reconstructions.

#### Evolutionary scenario of regional endothermy in lamniforms

**Fig 5:**
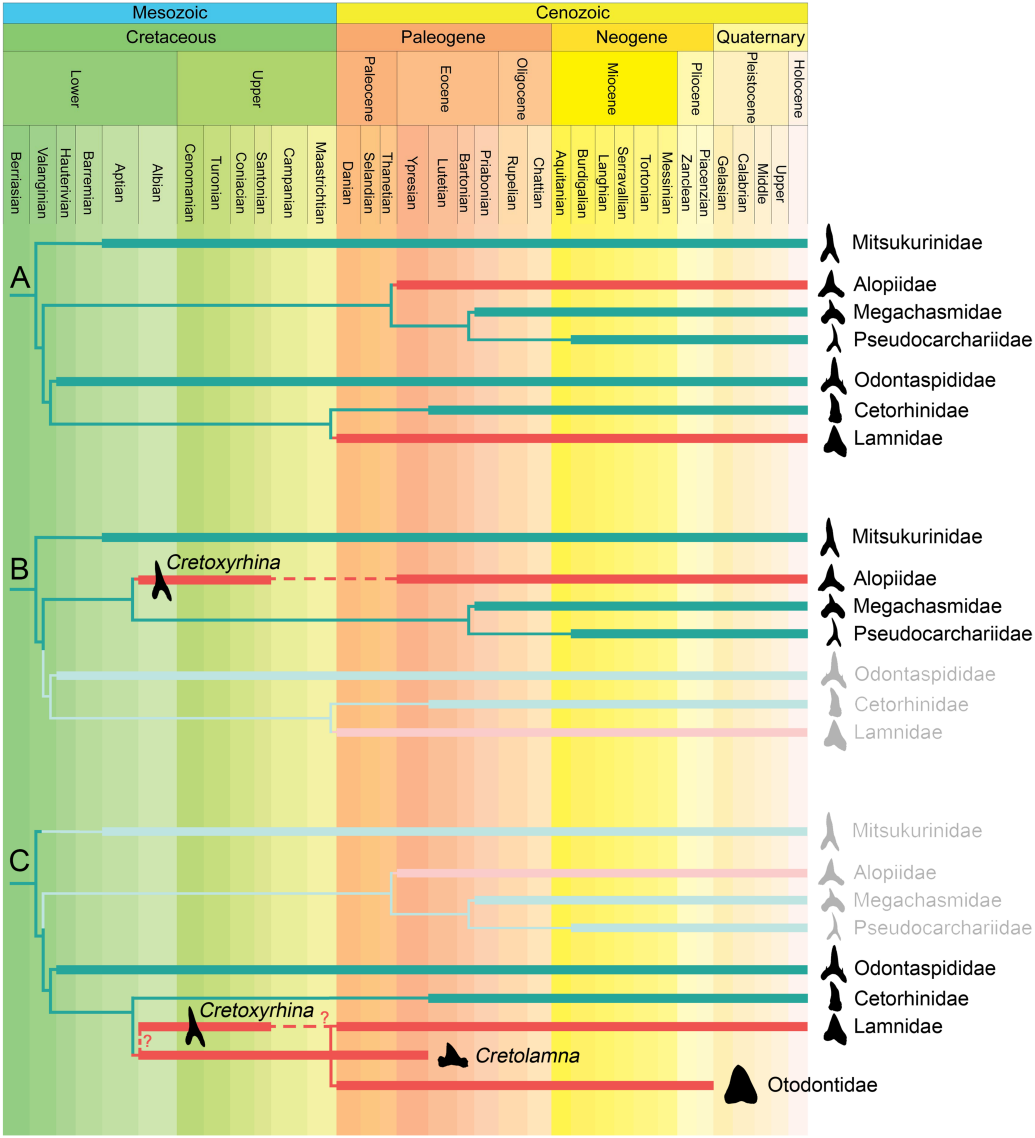
Possible evolutionary scenarios for the origin of regional endothermy in lamniforms considering (A) only extant lamniform groups, (B) *Cretoxyrhina* within alopids, and (C) *Cretolamna* representatives as ancestors of both lamnids and otodontids, and *Cretoxyrhina* within lamnids. Stratigraphic ranges taken from [1].

Among living fishes, endothermy is present in several orders and it is thought to have evolved independently at least six times (in lamnids, billfishes, the butterfly mackerel, tunas, alopids and lamprids [18,21,23]); but probably more if taking into account some other possible regional endotherms included within mobulids and labrids [164–166]. Here the presence of regional endothermy is strongly supported in two additional groups of extinct sharks, Cretoxyrhinidae and Otodontidae, pushing the appearance of this adaptation among fishes back to the Albian (Late Cretaceous) [1] (note that some authors [11,12] have also suggested the possible existence of endothermy in a few Paleozoic groups). Interestingly the evolution of regional endothermy in extant fish families is thought to occur much later, during the Eocene coinciding with a major cooling trend in the global climate ([18]; although see below). The monophyly of some cretoxyrhinids and otodontids is widely accepted (*Cretolamna*-otodontid lineage) [1,9,31,47,167–171], thus a common origin for the regional endothermy of both groups is the most parsimonious option. However, phylogenetic affinities between these extinct families and living lamniform groups are still controversial and, in consequence, it is difficult to establish the evolutionary relationship between the regional endothermy of cretoxyrhinids and otodontids and that of alopids and lamnids. Otodontids have usually been regarded as lamnids (e.g., [6,9,26–29]), as their sister-group, or as a closely related group (e.g., [1,5,30, 31–36]). In fact, both groups may have evolved from *Cretolamna* species, sharing a common ancestor within cretoxyrhinids [1,9,26,31]. At present, monophyly of cretoxyrhinids is not sustained and they are considered as a paraphyletic or even polyphyletic group although their systematics is maintained awaiting further results [1,172,173]. Cappetta [1] provisionally included five genera within this family, of which *Cretolamna* and *Cretoxyrhina* are the best known by far. The phylogenetic position of *Cretolamna* among the extant lamniform taxa remains largely unknown [44]. A number of works have proposed *Cretolamna* as being the “ancestor” of otodontids [1,9,31,47,167–171], *Cretoxhirhina* [1] and even of lamnids [1,9,26,31] on the basis of dental characters. Similarly, the phylogenetic position of *Cretoxyrhina* is also debated, having been included within cretoxhyrhinids traditionally [1], within alopids following the only cladistical approach on the group [174] and recently, after the discovery of one Cretaceous articulated specimen of *Isurus denticulatus*, *Cretoxyrhina* has been placed among the earliest species of the *Isurus* lineage as a lamnid [175]. In sum, several possible scenarios about the evolution of regional endothermy within lamniforms can be drawn (Fig 5), some of them implying the independent evolution of this adaptation up to three (or possibly more) times. Independent evolution of the regional endothermy of alopids seems to be clear on the basis of their phylogenetic position [176] and some morphological aspects of their orbital retia [17] (Fig 5A). Lower Eocene remains of *Alopias denticulatus* represent the earliest record of Alopiidae [173], however both the stratigraphic range and the origin of regional endothermy in alopiids could be extended back to the Late Cretaceous if *Cretoxyrhina* is included within the family (according to [174]) (Fig 5B). On the other hand, given the relatively close phylogenetic affinity between lamnids and otodontids, it is rather parsimonious to consider the regional endothermy of both groups to be homologous and have evolved at least during the Late Cretaceous (if including *Cretolamna* representatives as ancestors and/or considering *Cretoxyrhina* within *Isurus*; Fig 5C). However, different scenarios could be also possible and independent evolution of regional endothermy in cretoxyrhinids cannot be ruled out given the possible polyphyly and poorly resolved phylogenetic relationships of these groups. Therefore, the finding of further articulated remains that allow robust cladistic analyses (not only based on dental characters) are needed to unravel the affinities of otodontids and cretoxyrhinids and, in consequence, the origin of regional endothermy in lamniforms.

Several hypotheses have been proposed to explain the evolution of regional endothermy in living groups (see [18] for a detailed review). Among them, the thermal niche expansion hypothesis was supported by Dickson and Graham [18] comparing the thermal niche of extant taxa, their fossil record, and the paleoceanographic conditions during the time that endothermic fishes radiated. According to their ideas, oceanic cooling and tropical compression during the Eocene were the promoters of the almost simultaneous independent evolution of regional endothermy in several groups of fishes. However, the discovery of regional endothermy among cretoxyrhinids could extend the stratigraphic range of some regional endotherm lineages (e.g., lamnids and alopids) back to the Cretaceous thus calling into question this hypothesis. More recently, the elevated cruising speed hypothesis has gained support with the development of modern animal-tracking tools. In this sense, Watanabe et al. [85] have demonstrated in free swimming fishes that regional endotherms have higher cruise swimming speeds and exhibit larger-scale annual migrations than ectotherms. This increases prey encounter rates, enables larger-scale annual migrations and allows potentially greater access to seasonally available resources. The selection pressures that lead to the evolution of regional endothermy in cretoxyrhinids are difficult to ascertain. Current evidence only allows for speculation about this issue and for some possible scenarios to be suggested for testing in future studies. The thermal niche expansion hypothesis seems unlikely for this group as the first reports of both *Cretolamna* and *Cretoxyrhina* from the Albian (Late Cretaceous) [1] are coincident with an extremely warm climate and markedly reduced vertical and latitudinal temperature gradients (see for example [148,177]). On the other hand, the worldwide distributions of cretoxyrhinids [1] and morpho-functional interpretation of the caudal fin morphology of *Cretoxyrhina* (see above) support good migratory capacities and strong swimming capabilities compatible with the elevated cruising speed hypothesis. Also, regional endothermy could have provided cretoxyrhinids with several evolutionary advantages over their ectothermic prey and competitors ([18] and references therein). Interestingly, some groups of marine reptile predators underwent a rapid radiation and diversification during the Late Cretaceous [178,179] a few million years after the appearance of the first cretoxyrhinids [1]. Such evolutionary success has sometimes been explained by the acquisition of endothermy providing them with some advantages during the Late Cretaceous climate cooling [37,62]. Thus, the progressive cooling of the oceans [180] together with increasing competition with marine retile top-predators might also be suggested as major promoters of the evolution of regional endothermy in cretoxyrhinids (although see [181]). In any case, several selection pressures could have worked together to promote the evolution of regional endothermy in fishes and, at the same time, different evolutionary scenarios could be expected in each separate group.

Regarding the anatomical and physiological aspects of regional endothermy, diverse structures and mechanisms are involved in heat production and maintenance of high body temperatures in extant regional endothermic groups [18]. The physiological and anatomical adaptations that allowed high body temperatures in cretoxyrhinids and otodontids are difficult to determine. The large size and low surface area-to-volume ratio expected for some otodontids might suggest a passive accumulation of heat (gigantothermy). However, although this phenomenon could contribute to heat retention in these huge taxa, it seems unlikely that it played a major role in smaller forms such as *Cretolamna* where other active mechanisms are expected to be present. Considering then that *Cretolamna* occupies a basal position to (or within) otodontids [47], it is expected that such mechanisms were inherited by all the species of otodontids. On the other hand, the detection of relatively low δ^18^O values in teeth of cretoxirhinids and otodontids implies the existence of high temperatures in the anterior part of their bodies, consistent with the presence of cranial endothermy. Based on the apparent dependence of cranial endothermy on red muscle endothermy, Dickson and Graham [18] argued that the former should have evolved after or at the same time as the latter. Accordingly, regional endothermy could be present in lamniforms even earlier than proposed here. In this sense, performing isotopic analyses on postcranial endoskeletal elements (e.g., vertebra) could be interesting for testing the existence of other sources of heat within the trunk region, compatible with the presence of red muscle endothermy.

#### Distribution patterns, extinction and the evolution of gigantism in the megatooth sharks

The existence of an ectothermic physiology and/or low tolerance to cold waters in otodontids has sometimes been proposed as a constraining factor on their distribution patterns and as an underlying cause of their extinction [6,9,10,18]. Gottfried et al. [6] and Diedrich [10] suggested that differences in prey and water temperature preference in megatooth and white sharks led to the extinction of the former in the Pleistocene due to climate change and ocean cooling. Segregated distribution patterns of both taxa, with otodontids and white sharks inhabiting warmer and colder waters respectively, have been sometimes explained by competitive exclusion of the latter sharks from the preferred warmer areas of gigant-toothed species [9]. Similarly, Dickson and Graham [18] interpreted the differential success of white and megatooth sharks as a consequence of the differences in the thermal physiology of both taxa. Accordingly, the regional endothermy of white sharks allowed them to face oceanic cooling during the Pliocene-Pleistocene successfully, whereas some supposed ectothermic taxa such as *O*. *megalodon* became extinct. On the other hand, shifts in the distribution of large marine mammals to colder high-latitude conditions have also been proposed as possible causes of the extinction of otodontids [6]. However, Pimiento et al. [16] have recently demonstrated that the range of water temperatures inhabited by *O*. *megalodon* was wider than previously expected being rather consistent with the existence of regional endothermy in this taxon. Furthermore, they noted that the occupancy range of *O*. *megalodon* was not correlated with climatic changes thus suggesting that extinction of otodontids was not primarily driven by climatic change and ocean cooling. Results presented here strongly support the existence of regional endothermy in the *Cretolamna*-otodontid lineage making it necessary to integrate this phenomenon and its consequences (e.g., tolerance to a wider range of water temperatures and less vulnerability to ocean cooling) in new explanatory hypotheses about the extinction of otodontids. In this sense, hypotheses that involve global habitat loss produced by sea-level oscillations during the Pliocene [182] and/or biotic factors such as the drop in the diversity of potential prey (filter-feeding whales) or the appearance of new competitors (large predatory whales and the great white shark) could be more in agreement with all these aspects ([16,115] and references therein).

On the other hand, previous interpretations regarding the evolution and maintenance of gigantism in the biggest otodontids have been supported almost exclusively by the availability of high energetic blubber-rich marine mammals as prey. Gottfried et al. [6], based on the analysis of bite marks in fossil cetacean bones and previous dietary studies in extant white sharks, suggested that the gigant megatooth sharks could have relied on large blubber-rich marine mammals that may constituted an important part of their diet. Purdy [9] also noted that marine mammals could have constituted an important part of otodontid diet on the basis of positive correlation between the abundances of fossil remains of both groups. Regarding evolutionary intrinsic mechanisms that allowed gigantism in otodontids, Ehret [5] described acceleration of growth rates and delay in the offset timing of somatic growth in some otodontids but proposed again the evolution and diversification of cetaceans as the most plausible driver of these changes. However, although the availability of potential prey played a fundamental role in this regard, all these previous hypothesis obviated some other intrinsic aspects that should also be fundamental for maintaining an active lifestyle with such large body mass.

**Fig 6:**
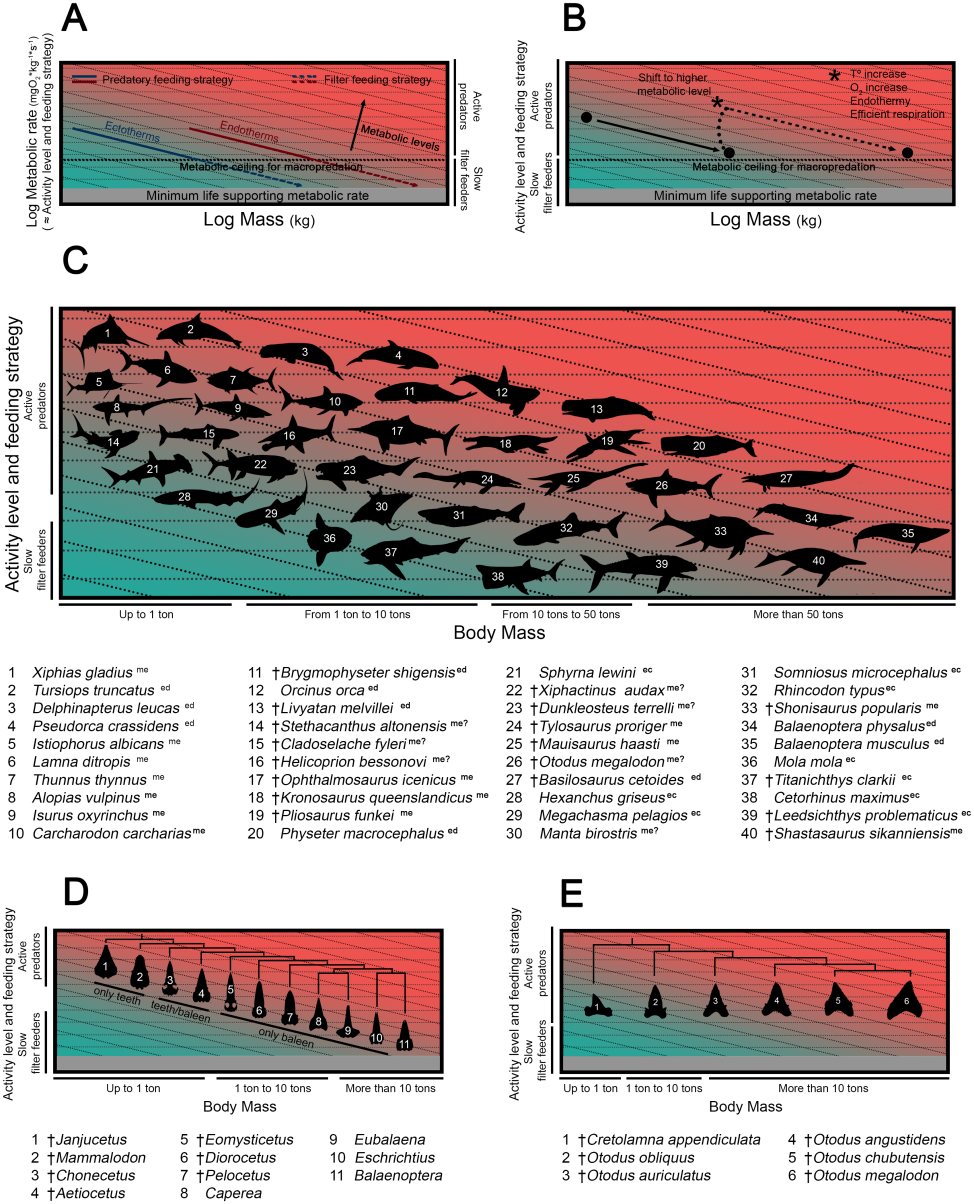
(A) Diagram showing the relationship between body mass, metabolic rate (≈ activity level and feeding strategy) and metabolic level (ecto, meso and endothermy) in aquatic vertebrates. (B) Schematic explanation of how shifts towards higher metabolic levels, promoted by different factors, contribute to maintaining a predatory lifestyle at bigger body sizes. (C) Diversity of body masses, feeding and thermoregulatory strategies of living and extinct aquatic vertebrates (ed, endotherm; ec, ectotherm; me, mesotherm; cross sign denotes an extinct taxon). A-C modified from Ferrón et al. [11]. (D-E) Diagrams showing body sizes, feeding and thermoregulatory strategies of mysticete cetaceans and otodontid sharks respectively. Mysticete skull and otodontid tooth outlines modified from Fitzgerald [185] and Pimiento and Balk [46]. Bluish and reddish tones represent lower and higher metabolic levels, respectively.

In this sense, Ferrón et al. [11] have recently provided key ideas to understand the evolution of gigantism in active vertebrate predators from a metabolic perspective. Activity and feeding strategies of living organisms are limited by specific values of mass-specific metabolic rate (i.e., metabolic ceilings). Given that mass-specific metabolic rate decreases when the body mass increases, active macropredation is not sustainable once a given body mass is reached and only less active modes of life and feeding strategies (e.g. filter feeding) are physiologically affordable above this size. However, this limit is reached at different body masses depending on thermoregulatory strategy and, ultimately, metabolic level; endothermic macropredators can attain larger potential body masses than their ectothermic counterparts (Fig 6A). Interestingly, shifts towards higher metabolic levels, promoted by different factors (i.e., increases in ambient temperature and atmospheric oxygen levels, evolution of highly efficient respiratory systems or acquisition of endothermy), can allow the same activity level and feeding strategy to be sustained at larger body masses, offering a suitable explanation for the evolution of gigantism in active macropredators (see Ferrón et al. [11] for further discussion) (Fig 6B). Among these factors, endothermy seems to have played an important role in the evolution of gigantism in many extinct macropredatory groups including some dinosaurs [134,183,184], ichthyosaurs, plesiosaurs and mosasaurs [37,62]. Ferrón et al. [11] also suggested that a number of extinct macropredatory fishes, including some members of the family Otodontidae, could have been regional or whole endotherms considering their presumed active lifestyle and big body sizes (Fig 6C). Now, regional endothermy of otodontids is strongly supported here from multiple approaches, and is confirmed as the key element that promoted the metabolic shift needed to reach huge sizes as macropredators in this group. Therefore, the integration of ecological and physiological triggers (i.e., availability of blubber-rich prey and endothermy inherited from cretoxyrhinids) offers a more holistic hypothesis to explaining the evolution of gigantism and the maintenance of active modes of life in this shark lineage.

The gigantic sizes reached by some otodontids were the result of an evolutionary tendency towards larger body sizes [46]. According to the ideas presented in Ferrón et al. [11], a trend to less active lifestyles in bigger species would be expected if all otodontids shared the same metabolic level. This phenomenon is well documented in the evolution of mysticetes where extinct small basal forms were actively carnivorous whereas more derived giant species are slow filter feeders (and intermediate-sized forms were probably facultative filter feeders) [185] (Fig 6D). However, the existence of similar active macropredatory lifestyles in all species of otodontids implies that more derived and larger forms would have had higher metabolic levels and, therefore, the tendency towards gigantism should be accompanied by a tendency towards a better-developed endothermy (Fig 6E). The factors that promoted this tendency towards gigantism are poorly understood. Pimiento and Balk [46] have recently argued that this phenomenon could be the result of a long-term selective pressure on otodontids favouring bigger forms with a broader range of prey. On the other hand, the fact that otodontids grew in size proportionally to cetaceans during the Neogene [10] could suggest that gigantism coevolved in both groups as a result of an evolutionary race between predators and prey. In either case, the acquisition of anatomical structures and physiological mechanisms that allowed regional endothermy in cretoxyrhinids probably played a key role in the subsequent evolutionary history of the lineage, acting as a trigger for the evolution of gigantism in more derived otodontids, allowing active macropredatory lifestyles and helping them in the struggle for survival.

## Supporting information

S1 Table Detailed δ^18^O data of cretoxyrhinids-otodontids and associated ectotherms compiled for this study. (XLSX)

S2 Table Simultaneous records of internal body organs/tissues and surrounding water temperature in three extant species of regional endothermic sharks. Data from Lowe and Goldman [7].
(XLSX)

S3 Table δ^18^O tooth values of cretoxyrhinids and ectothermic fishes from several Campanian-Maastrichtian localities and latitudes. Data from Pucéat et al. [6].
(XLSX)

S4 Table Records of cruise and burst swimming speed of living ectothermic and regional endothermic fishes. Standard Lengths (SL) and Fork Lengths (FL) has been transformed into Total Lengths (TL) using equations from Froese and Pauly [8] (equations from Kohler et al. [9] were also used but only for the case of *Isurus oxyrhinchus*). Records denoted with an asterisk were not considered for estimating swimming speeds of cretoxyrhinids and otodontids as these data and the methodology by which they were obtained have been sometimes questioned (see Results and discussion section).
(XLSX)

S5 Table Cobb's angle, hypochordal ray angle and aspect ratio measurements of several living lamniform shark species and the fossil taxon *Cretoxyrhina mantelli* (data from Kim et al. [83]). (XLSX)

S6 Table Routine metabolic rate (RMR) of several living ectothermic and regional endothermic sharks and rays adjusted to five different temperatures (5°C, 10°C, 15°C, 20°C, and 25°C). (XLSX)

S7 Table Records of cruise swimming speeds, water temperatures and body masses of living ectothermic and regional endothermic sharks. Data taken from Watanabe et al. [17].
(XLSX)

S1 Fig (A) Caudal fin variables used for aspect ratio inferences of *Cretoxyrhina mantelli*. Cobb’s angle (Ca) describes the curvature of the vertebral column segment comprised between the anterior-most caudal vertebra (acv) and the posterior caudal vertebra (pcv); Hypochordal Ray angle (HRa) describes the orientation of the longest hypochordal ray of the caudal fin (diagram modified from Kim et al. [83]). (B) Photographs of the well-preserved *C*. *mantelli* specimen CMN 40906 from Shimada et al. [84]: figs. 1 and 4; courtesy of the New Mexico Museum of Natural History & Science). Scale bar equals to 30 cm and 10 cm in the complete and enlarged view of the specimen respectively.
(TIF)

S2 Fig Power-performance curves relating the oxygen consumption (MO2, mgO2 kg-1 h-1) to relative swimming speed (U, l·s-1) of (A) *Carcharhinus acronotus* (from Carlson et al. [85]) and (B) *Isurus oxyrinchus* (from Sepulveda et al. [86]), used in this study as models of ectothermic and regional endothermic sharks for metabolic inferences in *Cretoxyrhina mantelli*. NCS, Net cost of swimming; SMR, Standard metabolic rate; TMR, Total metabolic rate.
(TIF)

S1 Text Power performance curves of small sharks as a tool for assessing the metabolic rate of bigger species. (DOCX)

S2 Text Aspect ratio and swimming speed inferences in *Cretoxyrhina*. (DOCX)

S3 Text References of supplementary information. (DOCX)
