# Supplementary figures and images for: Regional endothermy as a trigger for gigantism in some extinct macropredatory sharks
Source: PLoS One. 2017 Sep 22;12(9):e0185185. doi: 10.1371/journal.pone.0185185 (PMC5609766; doi:10.1371/journal.pone.0185185)

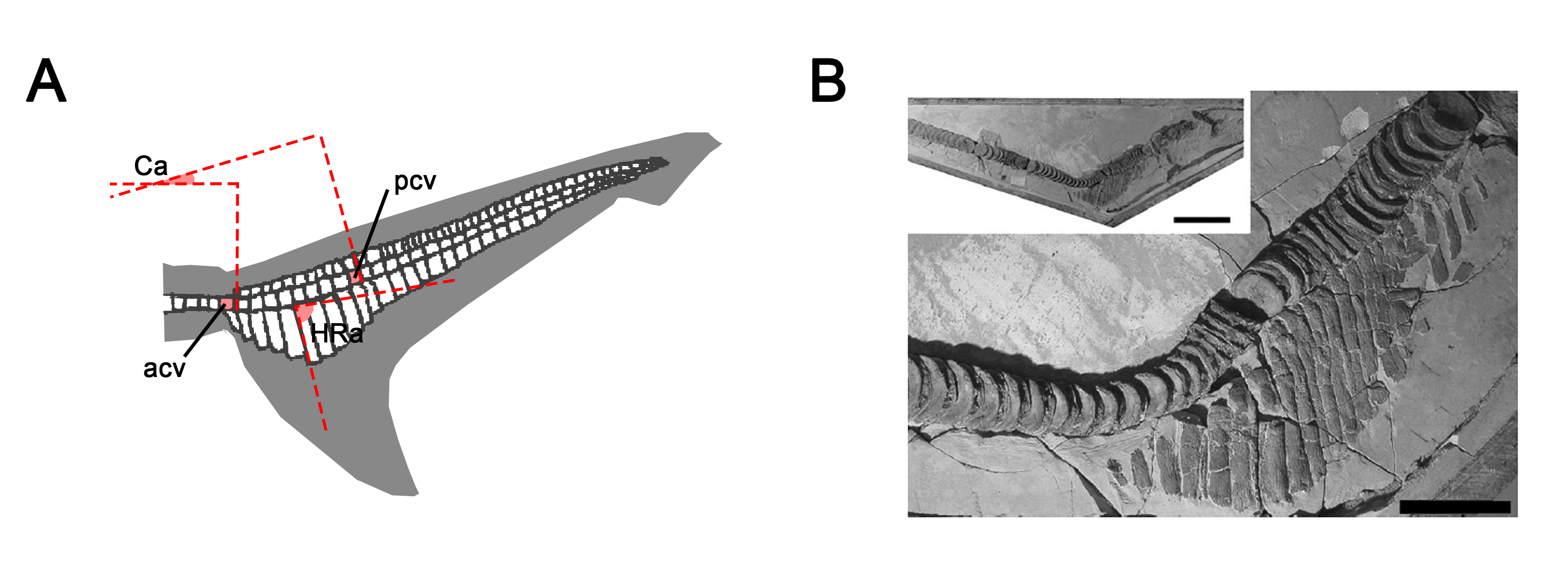

Supplement: S1 Fig — (A) Caudal fin variables used for aspect ratio inferences of Cretoxyrhina mantelli. Cobb’s angle (Ca) describes the curvature of the vertebral column segment comprised between the anterior-most caudal vertebra (acv) and the posterior caudal vertebra (pcv); Hypochordal Ray angle (HRa) describes the orientation of the longest hypochordal ray of the caudal fin (diagram modified from Kim et al. [83]). (B) Photographs of the well-preserved C. mantelli specimen CMN 40906 from Shimada et al. [84]: figs. 1 and 4; courtesy of the New Mexico Museum of Natural History & Science). Scale bar equals to 30 cm and 10 cm in the complete and enlarged view of the specimen respectively. (TIF) [file pone.0185185.s008.tif]

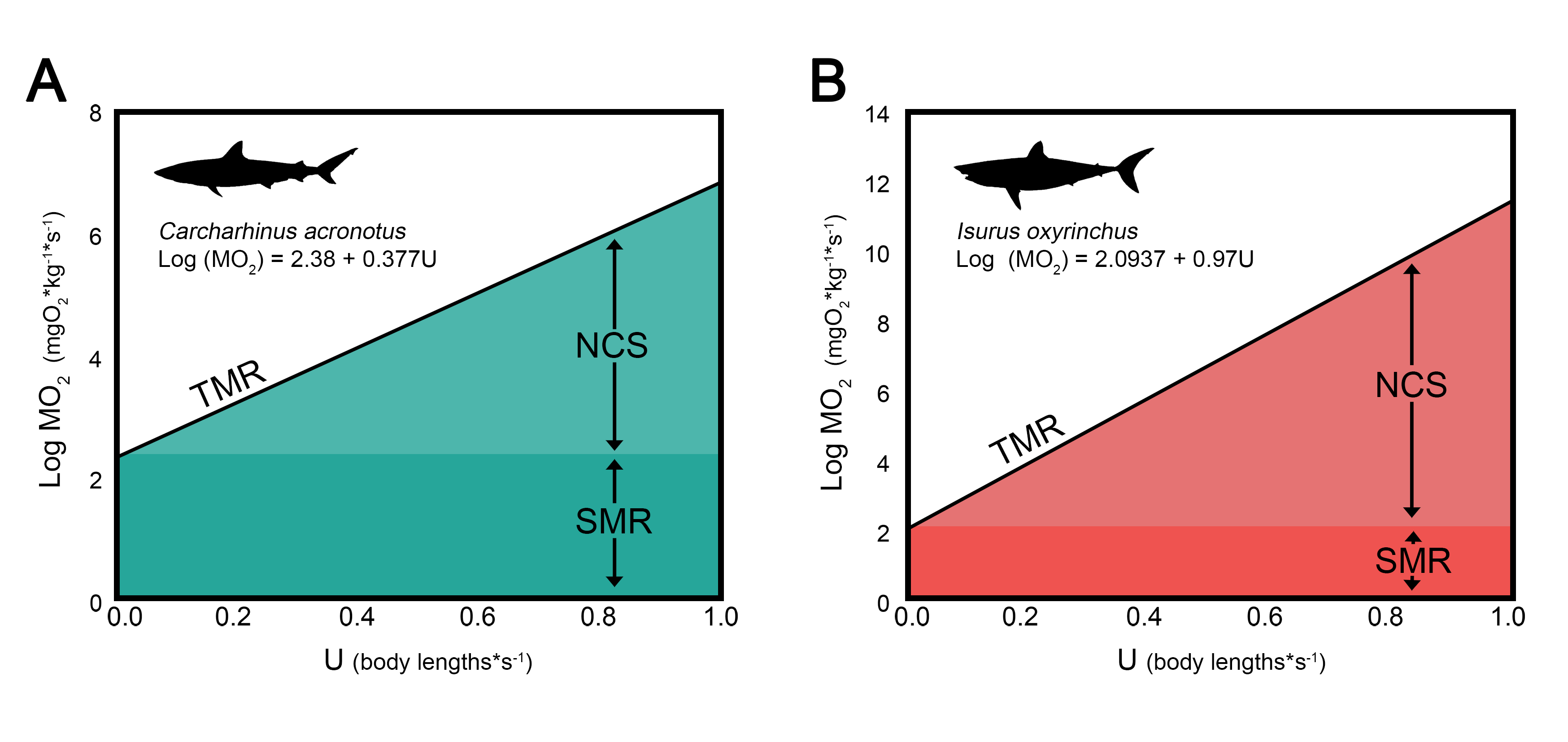

Supplement: S2 Fig — Power-performance curves relating the oxygen consumption (MO2, mgO2 kg-1 h-1) to relative swimming speed (U, l·s-1) of (A) Carcharhinus acronotus (from Carlson et al. [85]) and (B) Isurus oxyrinchus (from Sepulveda et al. [86]), used in this study as models of ectothermic and regional endothermic sharks for metabolic inferences in Cretoxyrhina mantelli. NCS, Net cost of swimming; SMR, Standard metabolic rate; TMR, Total metabolic rate. (TIF) [file pone.0185185.s009.tif]
