# Supplementary material for: Regional endothermy as a trigger for gigantism in some extinct macropredatory sharks
Source: PLoS One. 2017 Sep 22;12(9):e0185185. doi: 10.1371/journal.pone.0185185 (PMC5609766; doi:10.1371/journal.pone.0185185)
Supplement: S1 Text — (DOCX) [file pone.0185185.s010.docx]

Power-performance slopes (i.e., NCT) reported in sharks are very similar for all species studied so far and extremely similar for species with comparable lifestyles (active pelagic species usually show slightly higher slope values than the more sedentary demersal species). Comparable slopes have also been obtained for a relatively wide range of sizes, including sharks whose weights varied from 0.5 to 7.0 kg ([87]: table 4). Furthermore, NCT seem to be independent of the temperature (contrary to TMR), which simplifies their extrapolation to different scenarios regardless of this factor [88-90] (although see also [91]). This altogether suggests that, until further studies of oxygen consumption are not made on large sharks by means of more advanced techniques (e.g., [92]), power-performance curves of small species calculated in conventional respirometers could be suitable for assessing the metabolic rates of bigger species (always trying to select taxa with similar lifestyles and close phylogenetic affinity whenever possible). In fact, Semmens et al. [62] observed that the field metabolic rate of a 428 kg great white shark, inferred from power-performance curves of a 3.9 kg shortfin mako (*Isurus oxyrinchus*), fits properly with the mean food ration of captive individuals of the same species. On the other hand, NCT differ between regional endotherms and ectotherms, showing notably higher slopes in the former [93]. Here, considering all shark power-performance curves available in the literature, equations of *Carcharhinus acronotus* [85] and *Isurus oxyrinchus* [86] have been selected as the most suitable models for assessing the NCT of *Cretoxyrhina*, being good examples of active pelagic sharks with ectothermic and regional endothermic metabolism respectively.
