# Supplementary material for: Regional endothermy as a trigger for gigantism in some extinct macropredatory sharks
Source: PLoS One. 2017 Sep 22;12(9):e0185185. doi: 10.1371/journal.pone.0185185 (PMC5609766; doi:10.1371/journal.pone.0185185)
Supplement: S2 Text — (DOCX) [file pone.0185185.s011.docx]

Precise determination of *Cretoxyrhina* cruise swimming speed is a first crucial step for evaluating the net cost of swimming (NCS) of this taxon. In this sense, caudal fin morphology is clearly correlated with swimming capabilities in groups where the tail is the main structure involved in trust generation (body-caudal fin propulsion) [99]. As a consequence, some works have tried to establish reliable models for predicting swimming parameters in fishes from metric variables of the body and caudal fin (e.g., [18,100,101]). Here, *Cretoxyrhina* cruise (but also burst) swimming speed has been inferred following Sambilay’s [18] model mainly based on caudal fin aspect ratio and fish body length. Some large complete specimens of *Cretoxhyrhina*, found in Niobrara Chalk of western Kansas (USA), have allowed fairly accurate endoskeletal reconstructions and total body length estimations in this shark [84,102]. Specimen CMN 40906, preserving the posterior half of the vertebral column, has provided the most credible knowledge about the caudal fin morphology of *Cretoxyrhina* [84] (S5 Fig B). However, ventral lobe is lacking and, as consequence, aspect ratio cannot be directly measured. Despite that, the good correlation found between the aspect ratio of extant lamniform sharks and some other variables present on specimen CMN 40906 (Cobb’s angle and hypochordal ray angle) has allowed to infer the aspect ratio of *Cretoxyrhina* (See Fig 3). Estimation from hypochordal ray angle provides a value of 4.3 which has been considered here for subsequent steps as a conservative estimate; however a higher aspect ratio (around 4.9) is predicted according to the Cobb’s angle. These estimates represent by far the highest aspect ratios among lamniform sharks (See Fig 3C), being comparable to those of some scombrids [18], and suggest even higher swimming capabilities than the most active lamnid sharks. In fact, cruise and burst swimming speeds of 12 km*h^-1^ (0.53 body lengths*s^-1^) and 70 km*h^-1^ (3.04 body lengths*s^-1^) are predicted when applying Sambilay’s [18] model, being notably above the highest records of lamniforms in absolute terms (8.1 km*h^-1^ and 42.8 km*h^-1^ in *Carcharodon carcharias* S4 Table). In any case, an adaptation to extremely fast swimming is also supported by different morphofunctional aspects of the endoskeleton and the squamation (see main text).
