# Supplementary material for: Regional endothermy as a trigger for gigantism in some extinct macropredatory sharks
Source: PLoS One. 2017 Sep 22;12(9):e0185185. doi: 10.1371/journal.pone.0185185 (PMC5609766; doi:10.1371/journal.pone.0185185)
Supplement: S3 Text — (DOCX) [file pone.0185185.s012.docx]

1. Amiot R, Göhlich UB, Lécuyer C, De Muizon C, Cappetta H, Fourel F, et al. Oxygen isotope compositions of phosphate from Middle Miocene–Early Pliocene marine vertebrates of Peru. Palaeogeogr Palaeoclimatol Palaeoecol. 2008;264: 85–92.

2. Lécuyer C, Grandjean P, O’Neil JR, Cappetta H, Martineau F. Thermal excursions in the ocean at the Cretaceous—Tertiary boundary (northern Morocco): δ^18^O record of phosphatic fish debris. Palaeogeogr Palaeoclimatol Palaeoecol. 1993;105: 235–243.

3. Bernard A, Lécuyer C, Vincent P, Amiot R, Bardet N, Buffetaut E, et al. Regulation of body temperature by some Mesozoic marine reptiles. Science. 2010;328: 1379–1382.

4. Kocsis L, Gheerbrant E, Mouflih M, Cappetta H, Yans J, Amaghzaz M. Comprehensive stable isotope investigation of marine biogenic apatite from the late Cretaceous–early Eocene phosphate series of Morocco. Palaeogeogr Palaeoclimatol Palaeoecol. 2014;394: 74–88.

5. Pucéat E, Lécuyer C, Sheppard SM, Dromart G, Reboulet S, Grandjean P. Thermal evolution of Cretaceous Tethyan marine waters inferred from oxygen isotope composition of fish tooth enamels. Paleoceanography. 2003;18: 1–11.

6. Pucéat E, Lécuyer C, Donnadieu Y, Naveau P, Cappetta H, Ramstein G, et al. Fish tooth δ^18^O revising Late Cretaceous meridional upper ocean water temperature gradients. Geology. 2007;35: 107–110.

7. Lowe CG, Goldman KJ. Thermal and bioenergetics of elasmobranchs: bridging the gap. Environmental Physiology of Fishes. 2001;60: 251–266.

8. Froese R, Pauly D. FishBase. World Wide Web electronic publication. Version (04/2013). 2013. www.fishbase.org.

9. Kohler NE, Casey JG, Turner PA. Length-length and length-weight relationships for 13 shark species from the western north atlantic. National Marine Fisheries Service, 1996.

10. Kelly JT, Klimley AP, Crocker CE. Movements of green sturgeon, *Acipenser medirostris*, in the San Francisco Bay estuary, California. Environmental Biology of Fishes. 2007;79: 281–295.

11. Watanabe YY, Wei Q, Du H, Li L, Miyazaki N. Swimming behavior of Chinese sturgeon in natural habitat as compared to that in a deep reservoir: preliminary evidence for anthropogenic impacts. Environmental biology of fishes. 2013;96: 123–130.

12. Leonard JBK, Norieka JF, Kynard B, McCormick SD. Metabolic rates in an anadromous clupeid, the American shad (*Alosa sapidissima*). Journal of Comparative Physiology B: Biochemical, Systemic, and Environmental Physiology. 1999;169: 287–295.

13. Castro-Santos T. Optimal swim speeds for traversing velocity barriers: an analysis of volitional high-speed swimming behavior of migratory fishes. Journal of Experimental Biology. 2005;208: 421–432.

14. Taylor JC, Rand PS, Jenkins J. Swimming behavior of juvenile anchovies (Anchoa spp.) in an episodically hypoxic estuary: implications for individual energetics and trophic dynamics. Marine Biology. 2007;152: 939–957.

15. Ryan LA, Meeuwig JJ, Hemmi JM, Collin SP, Hart NS. It is not just size that matters: shark cruising speeds are species-specific. Marine Biology. 2015;162: 1307–1318.

16. McKibben JN, Nelson DR. Patterns of movement and grouping of gray reef sharks*, Carcharhinus amblyrhynchos*, at Enewetak, Marshall Islands. Bulletin of Marine Science. 1986;38: 89–110.

17. Watanabe YY, Goldman KJ, Caselle JE, Chapman DD, Papastamatiou YP. Comparative analyses of animal-tracking data reveal ecological significance of endothermy in fishes. Proceedings of the National Academy of Sciences. 2015;112: 6104–6109.

18. Sambilay VC. Interrelationships between swimming speed, caudal fin aspect ratio and body length of fishes. Fishbyte. 1990;8: 16–20.

19. Weihs D, Keyes RS, Stalls DM. Voluntary swimming speeds of two species of large carcharhinid sharks. Copeia. 1981;1981: 219–222.

20. Huish MT, Benedict C. Sonic tracking of dusky sharks in the Cape Fear River, North Carolina. Journal of the Elisha Mitchell Scientific Society. 1977;93: 21–26.

21. Medved RJ, Marshall JA. Short-term movements of young sandbar sharks, *Carcharhinus plumbeus* (Pisces, Carcharhinidae). Bulletin of Marine Science. 1983;33: 87–93.

22. Paig-Tran EM, Bizzarro JJ, Strother JA, Summers AP. Bottles as models: predicting the effects of varying swimming speed and morphology on size selectivity and filtering efficiency in fishes. Journal of Experimental Biology. 2011;214: 1643–1654.

23. Priede IG, Miller PI. A basking shark (*Cetorhinus maximus*) tracked by satellite together with simultaneous remote sensing II: new analysis reveals orientation to a thermal front. Fisheries Research. 2009;95: 370–372.

24. Harden-Jones FR. Tail beat frequency, amplitude, and swimming speed of a shark tracked by sector scanning sonar. Journal du Conseil. 1973;35: 95–97.

25. Gore MA, Rowat D, Hall J, Gell FR, Ormond RF. Transatlantic migration and deep mid-ocean diving by basking shark. Biology letters. 2008;4: 395–398.

26. Priede IG. A basking shark (*Cetorhinus maximus*) tracked by satellite together with simultaneous remote sensing. Fisheries Research. 1984;2: 201–216.

27. Sims DW. Filter-feeding and cruising swimming speeds of basking sharks compared with optimal models: they filter-feed slower than predicted for their size. Journal of Experimental Marine Biology and Ecology. 2000;249: 65–76.

28. Tudorache C, Viaenen P, Blust R, De Boeck G. Longer flumes increase critical swimming speeds by increasing burst–glide swimming duration in carp Cyprinus carpio, L. Journal of Fish Biology. 2007;71: 1630–1638.

29. Nakamura I, Meyer CG, Sato K. Unexpected positive buoyancy in deep sea sharks, *Hexanchus griseus*, and a *Echinorhinus cookei*. PloS one. 2015;10: e0127667.

30. Dutil JD, Sylvestre EL, Gamache L, Larocque R, Guderley H. Burst and coast use, swimming performance and metabolism of Atlantic cod *Gadus morhua* in sub‐lethal hypoxic conditions. Journal of Fish Biology. 2007;71: 363–375.

31. Watanabe YY, Lydersen C, Fisk AT, Kovacs KM. The slowest fish: swim speed and tail-beat frequency of Greenland sharks. Journal of Experimental Marine Biology and Ecology. 2012;426: 5–11.

32. Holland KN, Wetherbee BM, Lowe CG, Meyer CG. Movements of tiger sharks (*Galeocerdo cuvier*) in coastal Hawaiian waters. Marine Biology. 1999;134: 665–673.

33. Tricas TC, Taylor LR, Naftel G. Diel behavior of the tiger shark, *Galeocerdo cuvier*, at French Frigate Shoals, Hawaiian Islands. Copeia. 1981;1981: 904–908.

34. Heithaus MR, Wirsing AJ, Dill LM, Heithaus LI. Long-term movements of tiger sharks satellite-tagged in Shark Bay, Western Australia. Marine Biology. 2007;151: 1455–1461.

35. Westneat M, Walker J. Motor patterns of labriform locomotion: kinematic and electromyographic analysis of pectoral fin swimming in the labrid fish *Gomphosus varius*. Journal of Experimental Biology. 1997;200: 1881–1893.

36. Carey FG, Clark E. Depth telemetry from the sixgill shark, *Hexanchus griseus*, at Bermuda. Environmental Biology of Fishes. 1995;42: 7–14.

37. Behrens JW, Præbel K, Steffensen JF. Swimming energetics of the Barents Sea capelin (*Mallotus villosus*) during the spawning migration period. Journal of Experimental Marine Biology and Ecology. 2006;331: 208–216.

38. Nelson DR, McKibben JN, Strong WR, Lowe CG, Sisneros JA, Schroeder DM, et al. An acoustic tracking of a megamouth shark, *Megachasma pelagios*: a crepuscular vertical migrator. Environmental Biology of Fishes. 1997;49: 389–399.

39. Seymour RS, Farrell AP, Christian K, Clark TD, Bennett MB, Wells RM, et al. Continuous measurement of oxygen tensions in the air-breathing organ of Pacific tarpon (*Megalops cyprinoides*) in relation to aquatic hypoxia and exercise. Journal of Comparative Physiology B. 2007;177: 579–587.

40. Hartwell SI, Otto RG. Critical swimming capacity of the Atlantic silverside, *Menidia menidia* L. Estuaries and Coasts. 1991;14: 218–221.

41. Watanabe Y, Sato K. Functional dorsoventral symmetry in relation to lift-based swimming in the ocean sunfish *Mola mola*. 2008;PLoS One 3: e3446.

42. Barnett A, Abrantes KG, Stevens JB, Bruce BD, Semmens JM. Fine-scale movements of the broadnose sevengill shark and its main prey, the gummy shark. PloS one. 2010;5: e15464.

43. Campos BR, Fish MA, Jones G, Riley RW, Allen PJ, Klimley PA, et al. Movements of brown smoothhounds, *Mustelus henlei*, in Tomales Bay, California. Environmental biology of fishes. 2009;85: 3–13.

44. Graham JB, DeWar H, Lai NC, Lowell WR, Arce SM. Aspects of shark swimming performance determined using a large water tunnel. Journal of Experimental Biology. 1990;151: 175–192.

45. Gruber SH, Nelson DR, Morrissey JF. Patterns of activity and space utilization of lemon sharks, *Negaprion brevirostris*, in a shallow Bahamian lagoon. Bulletin of Marine Science. 1988;43: 61–76.

46. Sundström LF, Gruber SH. Using speed-sensing transmitters to construct a bioenergetics model for subadult lemon sharks, *Negaprion brevirostris* (Poey), in the field. Hydrobiologia. 1998;371: 241–247.

47. Hinch SG, Rand PS. Swim speeds and energy use of upriver-migrating sockeye salmon (*Oncorhynchus nerka*): role of local environment and fish characteristics. Canadian Journal of Fisheries and Aquatic Sciences. 1998;55: 1821–1831.

48. Kawabe, R, Naito Y, Sato K, Miyashita K, Yamashita N. Direct measurement of the swimming speed, tailbeat, and body angle of Japanese flounder (*Paralichthys olivaceus*). ICES Journal of Marine Science: Journal du Conseil. 2004;61: 1080–1087.

49. Pedersen J. Hydroacoustic measurement of swimming speed of North Sea saithe in the field. Journal of fish biology. 2001;58: 1073–1085.

50. Klimley AP, Beavers SC, Curtis TH, Jorgensen SJ. Movements and swimming behavior of three species of sharks in La Jolla Canyon, California. Environmental Biology of Fishes. 2002;63: 117–135.

51. Hueter RE, Tyminski JP, de la Parra R. Horizontal movements, migration patterns, and population structure of whale sharks in the Gulf of Mexico and northwestern Caribbean Sea. PLoS One. 2013;8: e71883.

52. Hsu HH, Joung SJ, Liao YY, Liu KM. Satellite tracking of juvenile whale sharks, *Rhincodon typus*, in the Northwestern Pacific. Fisheries Research. 2007;84: 25–31.

53. Rowat D, Meekan MG, Engelhardt U, Pardigon B, Vely M. Aggregations of juvenile whale sharks (*Rhincodon typus*) in the Gulf of Tadjoura, Djibouti. Environmental Biology of Fishes. 2007;80: 465–472.

54. Rowat D, Gore M. Regional scale horizontal and local scale vertical movements of whale sharks in the Indian Ocean off Seychelles. Fisheries Research. 2007;84: 32–40.

55. Eckert SA, Dólar BL, Kooyman GL, Perrin W, Rahman RA. Movements of whale sharks (*Rhincodon typus*) in South‐east Asian waters as determined by satellite telemetry. Journal of Zoology. 2002;257: 111–115.

56. Eckert SA, Stewart BS. Telemetry and satellite tracking of whale sharks, *Rhincodon typus*, in the Sea of Cortez, Mexico, and the north Pacific Ocean. Environmental Biology of Fishes. 2001;60: 299–308.

57. Gunn JS, Stevens JD, Davis TLO, Norman BM. Observations on the short-term movements and behaviour of whale sharks (*Rhincodon typus*) at Ningaloo Reef, Western Australia. Marine Biology. 1999;135: 553–559.

58. Lowe CG. Kinematics and critical swimming speed of juvenile scalloped hammerhead sharks. Journal of Experimental Biology. 1996;199: 2605–2610.

59. Holland KN, Wetherbee BM, Peterson JD, Lowe CG. Movements and distribution of hammerhead shark pups on their natal grounds. Copeia. 1993;2: 495–502.

60. Ackerman JT, Kondratieff MC, Matern SA, Cech JJ. Tidal influence on spatial dynamics of leopard sharks, *Triakis semifasciata*, in Tomales Bay, California. Environmental Biology of Fishes. 2000;58: 33–43.

61. Blake RW, Chan KHS, Kwok EWY. Finlets and the steady swimming performance of *Thunnus albacares*. Journal of fish biology. 2005;67: 1434–1445.

62. Semmens JM, Payne NL, Huveneers C, Sims DW, Bruce BD. Feeding requirements of white sharks may be higher than originally thought. Scientific reports. 2013;3: 1471.

63. Johnson R, Bester MN, Dudley SF, Oosthuizen WH, Meÿer M, Hancke L, et al. Coastal swimming patterns of white sharks (*Carcharodon carcharias*) at Mossel Bay, South Africa. Environmental Biology of Fishes. 2009;85: 189–200.

64. Bradford RW, Hobday AJ, Bruce BD. Regional Population Connectivity, Oceanic Habitat, and Return Migration Revealed by Satellite Tagging of White Sharks, *Carcharodon carcharias*, at New Zealand Aggregation Sites. In: Domeier ML, editor. Global Perspectives on the Biology and Life History of the White Shark. 2012. pp.301–318.

65. Bradford RW, Hobday AJ, Bruce BD. Identifying juvenile white shark behavior from electronic tag data. In: Domeier ML, editor. Global Perspectives on the Biology and Life History of the White Shark. 2012. pp.255–270.

66. Bradford RW, Hobday AJ, Bruce BD. Sex-Specific Migration Patterns and Sexual Segregation of Adult White Sharks, *Carcharodon carcharias*, in the Northeastern Pacific. In: Domeier ML, editor. Global Perspectives on the Biology and Life History of the White Shark. 2012. pp.133–146.

67. Bruce BD, Stevens JD, Malcolm H. Movements and swimming behaviour of white sharks (*Carcharodon carcharias*) in Australian waters. Marine Biology. 2006;150: 161–172.

68. Bruce, DB, Bradford RW. Spatial dynamics and habitat preferences of juvenile white sharks: identifying critical habitat and options for monitoring recruitment. CSIRO, 2008.

69. Bonfil R, Meÿer M, Scholl MC, Johnson R, O’brien S, Oosthuizen H, et al. Transoceanic migration, spatial dynamics, and population linkages of white sharks. Science. 2005;310: 100–103.

70. Bonfil R, Francis MP, Duffy C, Manning MJ, O’Brien S. Large-scale tropical movements and diving behavior of white sharks *Carcharodon carcharias* tagged off New Zealand. Aquatic Biology. 2010;8: 115–123.

71. Strong WR, Murphy RC, Bruce BD, Nelson DR. Movements and associated observations of bait-attracted white sharks, *Carcharodon carcharias*: a preliminary report. Marine and Freshwater Research. 1992;43: 13–20.

72. Carey FG, Kanwisher JM, Brazier O, Gabrielson G, Casey JG, Pratt Jr HL. Temperature and activities of a white shark, *Carcharodon carcharias*. Copeia. 1982;2: 254–260.

73. Goldman KJ, Anderson SD. Space utilization and swimming depth of white sharks, *Carcharodon carcharias*, at the South Farallon Islands, central California. Environmental Biology of Fishes. 1999;56: 351–364.

74. Sepulveda CA, Kohin S, Chan C, Vetter R, Graham JB. Movement patterns, depth preferences, and stomach temperatures of free-swimming juvenile mako sharks, Isurus oxyrinchus, in the Southern California Bight. Marine Biology. 2004;145: 191–199.

75. Carey FG, Teal JM, Kanwisher JW. The visceral temperatures of mackerel sharks (Lamnidae). Physiological Zoology. 1981;54: 334–344.

76. Holts DB, Bedford DW. Horizontal and vertical movements of the shortfin mako shark*, Isurus oxyrinchus*, in the Southern California Bight. Marine and Freshwater Research. 1993;44: 901–909.

77. Weng KC, Foley DG, Ganong JE, Perle C, Shillinger GL, Block BA. Migration of an upper trophic level predator, the salmon shark *Lamna ditropis*, between distant ecoregions. Marine Ecology Progress Series. 2008;372: 253–264.

78. Magnuson JJ. Locomotion by scombrid fishes: hydromechanics, morphology, and behavior. In: Hoar WS, Randall DJ, editors. Fish Physiology. Volume VII Locomotion. Vol. 7. 1979. pp.240–315.

79. Nakamura I, Watanabe YY, Papastamatiou YP, Sato K, Meyer CG. Yo-yo vertical movements suggest a foraging strategy for tiger sharks *Galeocerdo cuvier*. Marine Ecology Progress Series. 2011;424: 237–246.

80. Sundström LF, Gruber SH, Clermont SM, Correia JP, De Marignac JR, Morrissey JF, et al. Review of elasmobranch behavioral studies using ultrasonic telemetry with special reference to the lemon shark, *Negaprion brevirostris*, around Bimini Islands, Bahamas. Environmental Biology of Fishes. 2001;60: 225–250.

81. Martin RA, Hammerschlag N. Marine predator–prey contests: ambush and speed versus vigilance and agility. Marine Biology Research. 2012;8: 90–94.

82. Klimley AP, Le Boeuf BJ, Cantara KM, Richert JE, Davis SF, Van Sommeran S, et al. The hunting strategy of white sharks (*Carcharodon carcharias*) near a seal colony. Marine Biology. 2001;138: 617–636.

83. Kim SH, Shimada K, Rigsby CK. Anatomy and evolution of heterocercal tail in lamniform sharks. The Anatomical Record. 2013;296: 433–442.

84. Shimada K, Cumbaa SL, van Rooyen D. Caudal fin skeleton of the Late Cretaceous lamniform shark, *Cretoxyrhina mantelli*, from the Niobrara Chalk of Kansas. In: Lucas GS, Sullivan RM, editors. Late Cretaceous Vertebrates from the Western Interior. 2006. pp. 185–192.

85. Carlson, JK, Palmer CL, Parsons JR. Oxygen consumption rate and swimming efficiency of the blacknose shark, *Carcharhinus acronotus*. Copeia. 1999;1: 34–39.

86. Sepulveda CA, Graham JB, Bernal D. Aerobic metabolic rates of swimming juvenile mako sharks, *Isurus oxyrinchus*. Marine Biology. 2007;152: 1087–1094.

87. Dowd WW. Metabolic rates and bioenergetics of juvenile sandbar sharks (*Carcharhinus plumbeus*).The College of William and Mary Press, Virginia; 2003.

88. William F, Beamish H. Swimming metabolism and temperature in juvenile walleye, *Stizostedion vitreum vitreum*. Environmental Biology of Fishes. 1990;27: 309–314.

89. Claireaux G, Couturier C, Groison AL. Effect of temperature on maximum swimming speed and cost of transport in juvenile European sea bass (*Dicentrarchus labrax*). Journal of Experimental Biology. 2006;209: 3420–3428.

90. Ohlberger J, Staaks G, Hölker F. Effects of temperature, swimming speed and body mass on standard and active metabolic rate in vendace (*Coregonus albula*). Journal of Comparative Physiology B. 2007;177: 905–916.

91. Dickson KA, Donley JM, Sepulveda C, Bhoopat L. Effects of temperature on sustained swimming performance and swimming kinematics of the chub mackerel *Scomber japonicus*. Journal of Experimental Biology. 2002;205: 969–980.

92. Payne NL, Snelling EP, Fitzpatrick R, Seymour J, Courtney R, Barnett A, et al. A new method for resolving uncertainty of energy requirements in large water breathers: the “mega‐flume”seagoing swim‐tunnel respirometer. Methods in Ecology and Evolution. 2015;6: 668–677.

93. Bernal D, Carlson JK, Goldman KJ, Lowe CG. Energetics, metabolism, and endothermy in sharks and rays. In: Carrier CC, Musick JA, Heithaus MR, editors. Biology of Sharks and Their Relatives. 2012. pp. 211–237.

94. Carlson, JK, Parsons GR. Seasonal differences in routine oxygen consumption rates of the bonnethead shark. Journal of fish biology. 1999;55: 876–879.

95. Scharold J, Gruber SH. Telemetered heart rate as a measure of metabolic rate in the lemon shark, *Negaprion brevirostris*. Copeia. 1991;4: 942–953.

96. Brett JR, Blackburn JM. Metabolic rate and energy expenditure of the spiny dogfish, *Squalus acanthias*. Journal of the Fisheries Board of Canada. 1978;35: 816–821.

97. Ezcurra JM. The mass-specific routine metabolic rate of captive pelagic stingrays, *Dasyatis violacea*, with comments on energetics. California State University Press, Stanislaus; 2001.

98. Ezcurra JM, Lowe CG, Mollet HF, Ferry LA, O’Sullivan JB. Oxygen consumption rate of young-of-the-year white sharks, *Carcharodon carcharias*, during transport to the Monterey Bay Aquarium. In: Domeier ML, editor. Global Perspectives on the Biology and Life History of the White Shark. 2012. pp.17–26.

99. Lindsey CC. Form function locomotory habits in fish. In: Hoar HS, Randall DJ, editors. Fish Physiology. Volume VII Locomotion. 1979. pp. 1–88.

100. Fisher R, Hogan JD. Morphological predictors of swimming speed: a case study of pre-settlement juvenile coral reef fishes. Journal of Experimental Biology. 2007;210: 2436–2443.

101. Assumpção L, Makrakis MC, Makrakis S, Piana PA, da Silva PS, de Lima AF, et al. Morphological differentiation among migratory fish species from the Paraná River basin. Biota Neotropica. 2012;12: 41–49.

102. Shimada K. Skeletal anatomy of the Late Cretaceous lamniform shark, *Cretoxyrhina mantelli* from the Niobrara Chalk in Kansas. Journal of Vertebrate Paleontology. 1997;17: 642–652.
